# Non-small cell lung cancers (NSCLCs) oncolysis using coxsackievirus B5 and synergistic DNA-damage response inhibitors

**DOI:** 10.1038/s41392-023-01603-4

**Published:** 2023-09-25

**Authors:** Bopei Cui, Lifang Song, Qian Wang, Kelei Li, Qian He, Xing Wu, Fan Gao, Mingchen Liu, Chaoqiang An, Qiushuang Gao, Chaoying Hu, Xiaotian Hao, Fangyu Dong, Jiuyue Zhou, Dong Liu, Ziyang Song, Xujia Yan, Jialu Zhang, Yu Bai, Qunying Mao, Xiaoming Yang, Zhenglun Liang

**Affiliations:** 1 Division of Hepatitis and Enterovirus Vaccines, NHC Key Laboratory of Research on Quality and Standardization of Biotech Products, NMPA Key Laboratory for Quality Research and Evaluation of Biological Products, Institute of Biological Products, National Institutes for Food and Drug Control, Beijing, China; 2 National Engineering Technology Research Center for Combined Vaccines, Wuhan, China; 3 Beijing Minhai Biotechnology Co., Ltd, Beijing, China; 4 Taibang Biologic Group, Beijing, China; 5 Changchun Institute of Biological Products Co., Ltd, Changchun, China; 6 Shanghai Institute of Biological Products Co., Ltd, Shanghai, China; 7 China National Biotec Group Company Limited, Beijing, China

**Keywords:** Drug development, Lung cancer, Drug screening

## Abstract

With the continuous in-depth study of the interaction mechanism between viruses and hosts, the virus has become a promising tool in cancer treatment. In fact, many oncolytic viruses with selectivity and effectiveness have been used in cancer therapy. Human enterovirus is one of the most convenient sources to generate oncolytic viruses, however, the high seroprevalence of some enteroviruses limits its application which urges to exploit more oncolytic enteroviruses. In this study, coxsackievirus B5/Faulkner (CV-B5/F) was screened for its potential oncolytic effect against non-small cell lung cancers (NSCLCs) through inducing apoptosis and autophagy. For refractory NSCLCs, DNA-dependent protein kinase (DNA-PK) or ataxia telangiectasia mutated protein (ATM) inhibitors can synergize with CV-B5/F to promote refractory cell death. Here, we showed that viral infection triggered endoplasmic reticulum (ER) stress-related pro-apoptosis and autophagy signals, whereas repair for double-stranded DNA breaks (DSBs) contributed to cell survival which can be antagonized by inhibitor-induced cell death, manifesting exacerbated DSBs, apoptosis, and autophagy. Mechanistically, PERK pathway was activated by the combination of CV-B5/F and inhibitor, and the irreversible ER stress-induced exacerbated cell death. Furthermore, the degradation of activated STING by ERphagy promoted viral replication. Meanwhile, no treatment-related deaths due to CV-B5/F and/or inhibitors occurred. Conclusively, our study identifies an oncolytic CV-B5/F and the synergistic effects of inhibitors of DNA-PK or ATM, which is a potential therapy for NSCLCs.

## Introduction

The disability-adjusted life years of tumors were approximately 105 million years globally in 2019.^1^ Currently, immunotherapy is a hot spot for tumor treatment because of its targeting and security, including chimeric antigen receptor T-cell immunotherapy, immune checkpoint blockade, and oncolytic virus (OV).^2^ OVs are natural or genetically-modified viruses that can only recognize and lyse tumor cells, but cannot mire normal cells.^3^ For instance, T-vec was constructed to treat melanoma and was approved by the FDA in 2015.^4^ Moreover, many types of OVs, including DNA and RNA viruses, are in the research stage.^5^

The targeting and potency of an OV depend on many aspects. On the one hand, it relies on the expression of specific receptors on tumors but not on normal cells. However, signaling pathways can assist or compromise this effect. Furthermore, neutralizing antibody levels at baseline or primed by OV causes concerns of a limited “one shot” approach.^6,7^ Therefore, OV is required to be low seroprevalent and its definite receptor and oncolytic mechanism should be determined.

Recently, oncolytic enteroviruses have been reported to exert an oncolytic effect on tumor cells in a receptor-dependent manner, including coxsackievirus A21 (CV-A21), Coxsackievirus B3 (CV-B3), and Echovirus 7.^8–11^ For CV-A21, melanoma is the most sensitive cell type which is highly prevalent in Europe and America, and a phase II clinical trial has been launched.^8,12^ Enteroviruses appear to be a safer modality that replicates in the host cytosol without a DNA phase. As a result, they are unlike a DNA virus and lack the genotoxicity caused by the integration of the viral genome into the host DNA.^13^ Enteroviruses can utilize the internal ribosome entry site to conduct cap-independent translation.^14^ They can replicate rapidly, activate inflammatory responses, upregulate the expression of cytokines and chemokines, and induce anti-tumor immune responses. However, enteroviruses are the main pathogens causing hand-foot-and-mouth disease (HFMD) in infants and children, including Enterovirus A71, Coxsackievirus A16, Coxsackievirus A6, Coxsackievirus A10, CV-B3, Coxsackievirus B5 (CV-B5), Echovirus 30, and Echovirus 25, with a relatively high seroprevalence, which may be an obstacle in its application.^15^ Moreover, these common types of tumors in Asia, including lung cancer and liver cancer, have not been evenly emphasized in OV research. Thus, it is urgent to exploit more oncolytic enteroviruses that can be selected according to pre-existing neutralizing antibodies and used for highly prevalent cancers in Asia.

Although OV treatment has a relatively great effect on animal models, the therapeutic efficacy of OVs in clinical trials has not met the expectations based on preclinical models. Hence, the use of small molecules to selectively enhance the oncolytic effect and OV replication at tumor sites has proven to be a promising approach. For instance, PKA, VCP, or Bcl-xL inhibitors can synergize with the oncolytic M1 to selectively lyse tumors in vitro and in vivo.^16–18^ Nevertheless, no combinatorial inhibitors have been reported to synergize with oncolytic enteroviruses because of a lack of research on the oncolytic mechanism.

In this study, we aimed to study CV-B5/Faulkner (CV-B5/F) for its potential oncolytic effect against non-small cell lung cancers (NSCLCs), and provided a class of synergistic drugs that are beneficial for enhancing the curative effect on refractory cells.

## Results

### CV-B5/F is identified as an oncolytic candidate for NSCLCs

**Fig. 1:**
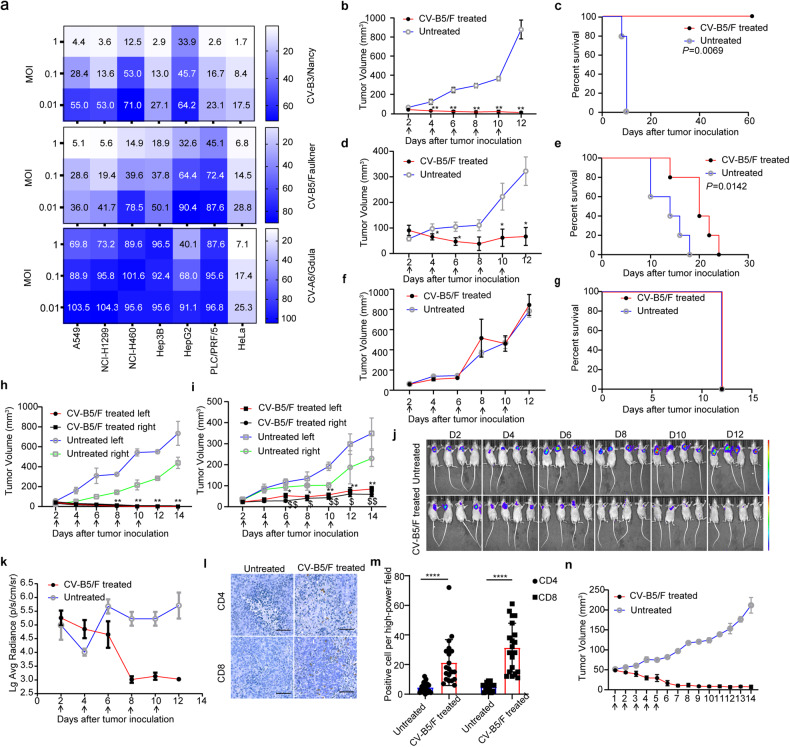
Screening of an oncolytic virus and its anti-tumor effects in multiple tumor models. **a** Non-small cell lung cancer cells (NSCLCs; A549, NCI-H1299, NCI-H460), hepatocarcinoma cells (Hep3B, HepG2, PLC/PRF/5), cervical carcinoma cells (HeLa) were infected with CV-B3/Nancy, CV-B5/Faulkner (CV-B5/F), CV-A6/Gdula at an MOI of 1, 0.1, 0.01 for 48 h. Cell viability was assessed by CCK8 assay. **b**–**g** NCI-H1299 (*n* = 5, **b**), A549 (*n* = 5, **d**), and NCI-H460 (*n* = 5, **f**) cells were subcutaneously injected into the right flank of BALB/c nude mice. Each mouse received five doses of CV-B5/F intratumorally when the diameter of the tumor reached 4–5 mm. Tumor volumes are expressed as mean ± SEMs. *P < 0.05; **P < 0.01. Kaplan–Meier survival analyses were performed for CV-B5/F-treated mice with five doses (**c**: NCI-H1299; **e**: A549; **g**: NCI-H460). **h**, **i** Administration of CV-B5/F to the right tumor (**h**: NCI-H1299, *n* = 5; **i**: A549, *n* = 5) was performed in BALB/c nude mice with bilateral tumors. *, $*P* < 0.05; **, $$*P* < 0.01. Each symbol represents the statistical significance of the right and left lateral tumors between untreated and treated mice. **j**, **k** NCI-H1299 cells expressing firefly luciferase were subcutaneously injected into the axillia of BALB/c nude mice (*n* = 4). The bioluminescent images were measured using the IVIS-Lumina II imaging system on days 2, 4, 6, 8, 10, and 12. Relative bioluminescence intensity is shown in pseudocolor, with red representing the strongest and blue representing the weakest photon fluxes (**j)**. The bioluminescence intensities of control and CV-B5/F-treated mice are shown as mean ± deviation (**k)**. **l**, **m** NCI-H1299 was combined with Matrigel at a 1:1 ratio immediately before subcutaneous injection into the axillia of B-NDG mice. Seven days after treatment, PBMCs were injected intraperitoneally in PBS. On day 10, tumors were dissected for immunohistochemistry (IHC) using anti-CD4 and CD8 antibodies. Numbers of positive cell in high-power field (*n* = 20) were analyzed. One-way ANOVA was used to analyze the data. Scale bars, 100 μm. ****P < 0.0001. **n** Tumor volume curves of patient-derived xenografts (PDX) for NSCLC after treatment with five doses of CV-B5/F

CV-B3/Nancy has been reported for its oncolytic effect on lung cancer, which evokes us to question whether CV-B5 can be a novel oncolytic virus.^9^ CCK8 experiments were used to compare and select the optimal strain for oncolysis in NSCLCs, hepatocarcinoma, and cervical cancer cell lines. CV-B3/Nancy, CV-B3/112, CV-B5/F, and CV-B5/JS417 eminently lysed all cell lines, while CV-A6 did not show oncolytic effects (Fig. 1a and Supplementary Fig. 1a). HeLa cells are sensitive to enterovirus propagation, which was set as control group.^19,20^ In liver cancer cell lines, the oncolytic effect of CV-B3 was similar to that of CV-B5 at the same dosage. In NSCLC cell lines, CV-B5 and CV-B3 showed robust oncolytic effects. Nevertheless, CV-B3 has been identified as an oncolytic virus in many types of cancers.^21^ Thus, the prototype strain CV-B5/F served as the optimal strain for oncolysis because no apparent differences were detected between these two strains of CV-B5.

Next, we evaluated the in vivo oncolytic effects of CV-B5/F in BALB/c nude mice bearing subcutaneous NSCLC cell derived xenografts (CDX). Five consecutive doses of CV-B5/F or CV-B5/JS417 were intratumorally administered into NCI-H1299, A549, and NCI-H460 xenografts at a 2-day interval. The results showed that treatment with CV-B5/F or CV-B5/JS417 elicited complete elimination of NCI-H1299 CDX and caused significant tumor regression in A549 CDX (Fig. 1b, d, Supplementary Fig. 1b, c), with significantly prolonged survival (Fig. 1c, e). However, treatment with CV-B5/F did not inhibit the growth of NCI-H460 xenografts (Fig. 1f, g). To further investigate the systemic oncolytic effects of CV-B5/F, we established bilateral CDX models of NCI-H1299 and A549 cells. Five consecutive administrations of CV-B5/F into the right flank significantly suppressed the growth of CV-B5/F-injected tumors and distant untreated tumors when compared with control (Fig. 1h, i). To evaluate the dose-dependent oncolytic effects, different dosages of CV-B5/F were used to treat NCI-H1299 CDXs (a volume of approximately 50 mm^3^ or 150 mm^3^). As shown in Supplementary Fig. 1d, e, 5/4/3 injections of CV-B5/F suppressed NCI-H1299 xenografts with a volume of approximately 150 mm^3^, all with significantly prolonged survival. Furthermore, 5/4/3/2/1 dosages of CV-B5/F completely eliminated NCI-H1299 xenografts with a volume of approximately 50 mm^3^ (Supplementary Fig. 1f). Notably, none of the mice died of side effects during these treatments, and histopathological evidence showed no obvious damage to the main organs (Supplementary Fig. 1g). To directly observe the elimination of NCI-H1299 xenografts, we implanted BALB/c nude mice with NCI-H1299 cells expressing firefly luciferase (luc). Moreover, the luminescence intensity of the tumors was reduced after treatment with CV-B5/F (Fig. 1j, k). Thus, CV-B5/F manifested good therapeutic effects on NSCLCs.

To investigate the tumor-infiltrating immune cells induced by intratumoral CV-B5/F administration, we established a humanized animal model based on *β2m* knockout B-NDG mice pre-injected with human peripheral blood mononuclear cells (PBMC) bearing NCI-H1299-luc xenografts and treated them with five consecutive injections of CV-B5/F.^22^ CV-B5/F administration suppressed tumor growth and increased the accumulation of CD3-, CD4-, CD8-, and Granzyme B positive cells (Fig. 1l, m, and Supplementary Fig. 1h- l). Together, CV-B5/F can activate adaptive immunity to enhance the oncolytic effect.

To simulate authentic tumors at maximum, we established patient-derived xenografts (PDX) in NPI mice. Five consecutive injections of CV-B5/F inhibited tumor growth (Fig. 1n and Supplementary Fig. 1m). Together, CV-B5/F showed oncolytic effects in vitro and in many kinds of animal models.

### Correlation between CV-B5/F-mediated oncolytic effects and the expression levels of surface receptors in cancer cell lines

**Fig. 2:**
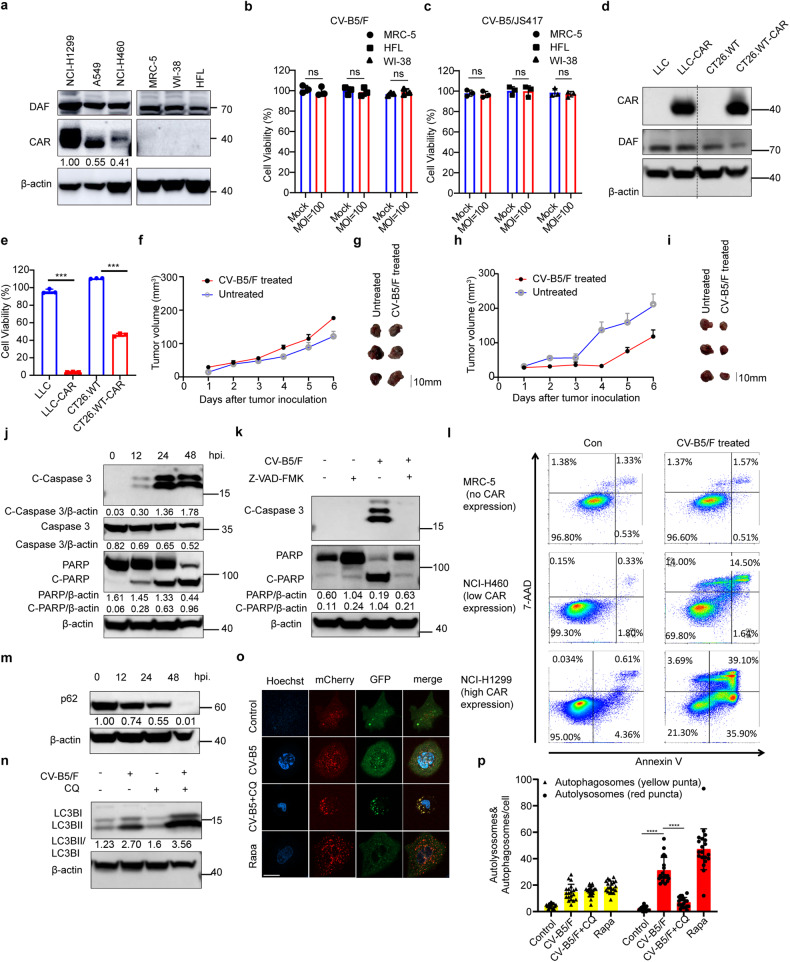
Expression profile of CAR and DAF on normal lung and cancer cells and its correlation with CVB5-mediated cytotoxicity. **a** Expression of CAR and DAF on normal lung cells (MRC-5, WI-38, and HFL) and NSCLC cells analyzed by western blot. Gray value ratios to the first lane of CAR were shown. **b**, **c** Normal lung cells infected with CV-B5/F (**b)** or CV-B5/JS417 (**c)** at 100 MOI and analyzed at 48 h for cell viability by CCK8 assay (*n* = 3). Each value represents the mean ± deviation. **d** Expression of CAR and DAF on wild-type or CAR over-expressing mouse Lewis lung cancer (LLC; LLC-CAR) and colorectal carcinoma (CT26.WT; CT26.WT-CAR). **e** LLC, LLC-CAR, CT26.WT, CT26.WT-CAR infected with CV-B5/F at 10 MOI was analyzed at 48 h for cell viability by CCK8 assay (*n* = 3). Each value represents the mean ± standard deviation. **f**–**i** LLC (**f**, **g)** or LLC-CAR (**h**, **i)** were subcutaneously injected into the axillia of C57BL/6 mice. Each mouse received 5 doses of CV-B5/F or with MEM intratumorally. Tumor were measured every day and anatomized ultimately (*n* = 3). **j** NCI-H1299 infected with CV-B5/F (MOI = 0.01) were analyzed at different time points. Each cellular lysate obtained was subjected to immunoblot analysis. Full-length PARP (116 kDa), cleaved-PARP (85 kDa), full-length caspase 3 (35 kDa) and cleaved-caspase 3 (17/19 kDa) were shown. hpi, hours post infection. **k** NCI-H1299 pretreated with 100 µM Z-VAD-FMK or MOCK and incubated with MEM or CV-B5/F at 0.01 MOI were subjected to immunoblot analysis. Full-length PARP (116 kDa), cleaved-PARP (85 kDa), and cleaved-caspase 3 (17/19 kDa) were shown. **l** NCI-1299, NCI-H460, and MRC-5 were infected with 1 MOI CV-B5/F for 24 h. Apoptotic population was represented as Annexin V ^+^/7-AAD^-^ or Annexin V^+/^7-AAD^+^ cells. **m** NCI-H1299 infected with CV-B5/F (MOI = 0.01) were analyzed at 0, 12, 24, and 48 h. p62 (62 kDa) was detected for the whole cell lysis (WCL). **n** NCI-H1299 was pretreated with 100 μM CQ for 2 h, and then treated with 0.01 MOI CV-B5/F for 24 h. LC3B (14/16 kDa) was detected for the WCL. **o,** **p** NCI-H1299 was transfected with mcherry-GFP-LC3B for 24 h, and then treated with 0.01 MOI CV-B5/F with or without 100 μM CQ (10 μM Rapamycin as positive control). Autophagosomes display both GFP and mCherry fluorescence (yellow-green), whereas autolysosomes display only mCherry fluorescence (red) because GFP is denatured by the acidity of the lysosome (**o)**. Number of autophagosomes and autolysosomes were enumerated for at least 20 cells (*n* = 20, **p**). One-way ANOVA was used to analyze the data. ***P < 0.001, ****P < 0.0001, ns, not significant. Scale bars, 10 μm

To assess the significant effects of receptor expression on cancer lines, we first detected the receptor expression and cell viability infected with CV-B5/F of the three NSCLC cell lines and three normal lung fibroblast cell lines, including MRC-5, WI-38, and HFL. The results indicated that these six cell lines expressed DAF; CAR was not expressed on normal lung cells but expressed on lung cancer cells with varying degrees (Fig. 2a and Supplementary Fig. 2a). CAR is the main receptor of CV-B5, and DAF is a co-receptor that assists entry.^23^ CV-B5/F and CV-B5/JS417 did not induce cytolysis in normal lung cells, even at an MOI of 100, which ensured the security of CV-B5 used for the treatment of lung cancer (Fig. 2b, c). In addition, we used a lentivirus to overexpress CAR and luc on mouse Lewis lung cancer cells (LLC) and mouse colorectal cancer cells (CT26.WT) (Fig. 2d). The results of the cytotoxicity assay indicated that LLC and CT26.WT cells overexpressing CAR were more sensitive to the oncolytic effects of CV-B5/F (Fig. 2e). The volumes of subcutaneous xenografts of LLC-CAR and CT26.WT-CAR were suppressed after five consecutive injections of CV-B5/F, and the counterpart wild-type xenografts showed no treatment effect (Fig. 2f–i, Supplementary Fig. 2b, 2d, e). Furthermore, injection of CV-B5/F induced CD3^+^CD4^+^ and CD3^+^CD8^+^ T cell infiltration (Supplementary Fig. 2c). Collectively, these findings indicated that CAR was essential for oncolysis and it formed a basis for the use of CV-B5/F in therapeutic applications for NSCLCs.

### Apoptosis and autophagy pathways are activated in NSCLCs following infection with CV-B5/F

To determine whether CV-B5/F (MOI = 0.1) induced apoptosis and autophagy in NSCLC cells, we examined cleaved-caspase 3, cleaved-PARP, LC3B, and p62. Western blot analysis revealed that NCI-H1299 and NCI-H460 cells showed time-dependent apoptosis (Fig. 2j and Supplementary Fig. 2f). In addition, we treated NCI-H1299 cells with a pan-caspase inhibitor before infected with CV-B5/F. We found that apoptosis was inhibited (Fig. 2k). Furthermore, we analyzed the externalization of phosphatidylserine and DNA fragmentation markers in cells infected with CV-B5/F, with or without CAR expression. The results indicated that NCI-H1299 (high CAR expression) and NCI-H460 (low CAR expression) cells showed prominent apoptosis after infection, and MRC-5 (without CAR expression) cells showed no apoptosis (Fig. 2l). In addition, we determined whether CV-B5/F could induce autophagy. Western blotting results indicated that CV-B5/F treatment reduced p62 levels and promoted LC3B lipidation (Fig. 2m). Furthermore, we used confocal microscopy to observe the autophagic flux induced by CV-B5/F. The results indicated that CV-B5/F induced significant autophagy in NCI-H1299 cells, which was inhibited by chloroquine (CQ) (Fig. 2n–p). These data demonstrated that apoptosis and autophagy were activated by the infection of CV-B5/F, which was the main pathway for oncolysis.

In the CDX model of NCI-H1299 in B-NDG mice, we used immunohistochemistry (IHC) to detect cleaved-caspase 3 and Ki67. The results indicated that intratumoral injection of CV-B5/F inhibited cell proliferation and enhanced caspase 3 cleavage (Supplementary Fig. 2g, h). The PDX model showed that CV-B5/F treatment drastically induced cleaved-caspase 3 and lipidated LC3B (Supplementary Fig. 2i). Thus, apoptosis and autophagy were also activated in vivo.

### DNA damage response inhibitors synergize with CV-B5/F to promote refractory cell death

**Fig. 3:**
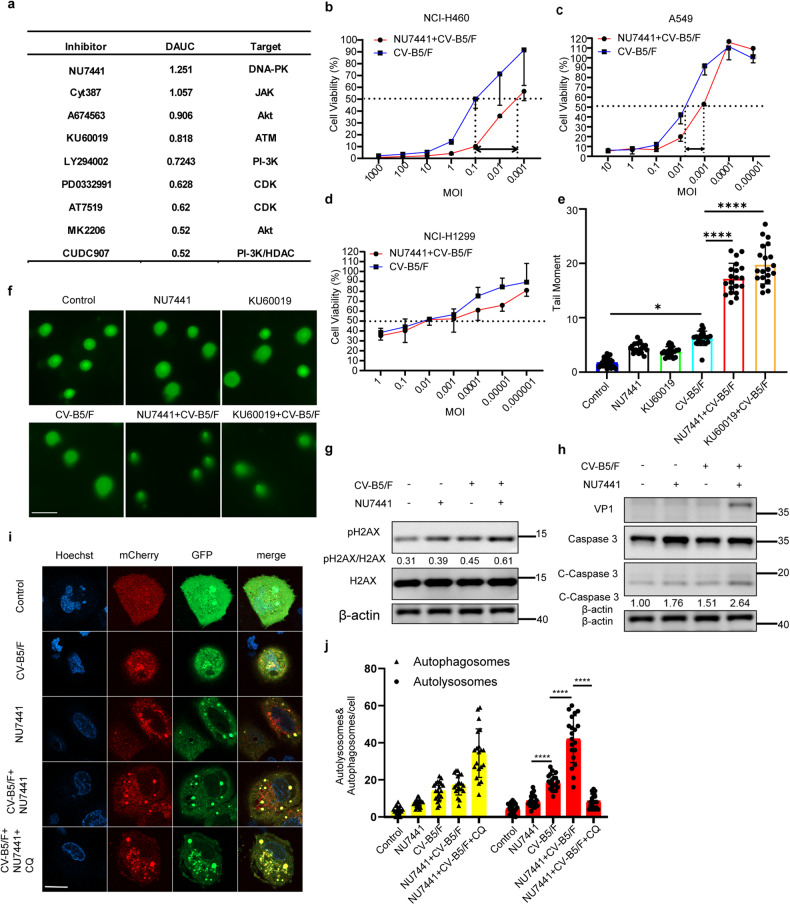
Combinatorial drug screen identifies DNA-PKI NU7441 and ATMI KU60019 as synergistic sensitizers for CV-B5/F in NSCLC cells. **a** NCI-H460 cells were seeded in 96-well plates and treated with escalating doses of each of the 30 compounds in the drug screen, either singly or in combination with CV-B5/F (MOI = 0.01). Top 9 candidate drugs for NCI-H460 identified through this screening. DNA-PK DNA-dependent protein kinase, JAK Janus kinase, Akt serine/threonine kinase, ATM ataxia telangiectasia mutated protein, PI3K phosphatidylinositol 3-kinase, CDK cyclin-dependent kinase, HDAC histone deacetylase. **b**–**d** NCI-H460, A549 and NCI-H1299 cells treated with escalating titers of CV-B5/F with or without 1 μM NU7441 for 48 h (*n* = 3). EC_50_ shifts were shown. **e**, **f** NCI-H460 cells were treated with NU7441 (1 μM), KU60019 (1 μM), CV-B5/F (MOI = 0.01), NU7441/CV-B5/F or KU60019/CV-B5/F for 24 h, and comet assay was used to assess double-strand breaks (DSBs). Quantification of tail moment was analyzed by OpenComet software (*n* = 20). Scale bar, 40 μm. **g** NCI-H460 cells were exposed to NU7441 (1 μM), CV-B5/F (MOI = 0.01) or a combination as indicated. p-H2AX (a marker of DNA damage response) was determined by western blot. **h** NCI-H460 cells were exposed to NU7441 (1 μM), CV-B5/F (MOI = 0.01) or a combination as indicated. Structural viral protein VP1 and cleaved-caspase 3 were determined by western blot. **i**, **j** NCI-H460 was transfected with mcherry-GFP-LC3B for 24 h, and then treated with 0.01 MOI CV-B5/F, 1 μM NU7441 or a combination with or without 100 μM CQ. The number of autophagosomes and autolysosomes were enumerated for 20 cells at least. One-way ANOVA was used to analyze data. *P < 0.05, ****P < 0.0001, Scale bar, 10 μm

To identify the potential mechanisms affecting oncolytic effects and screen synergistic drugs, we performed a combinatorial drug screening in refractory NCI-H460 cell lines using 30 agents that inhibit pathways involved in growth, metabolism, and apoptosis. Cell viability was assessed after treatment with increasing doses of drugs in the presence or absence of CV-B5/F.^17^ A low viral titer (0.01 MOI) was used so that the virus alone caused minimal cell death. The differences in area under the curve (DAUCs) for each compound with and without CV-B5/F were calculated (Fig. 3a and Supplementary Fig. 3a). We identified numerous compounds targeting DNA-dependent protein kinase (DNA-PK), Janus kinase (JAK), protein kinase B (PKB or Akt), ataxia telangiectasia mutated protein (ATM), phosphoinositide 3-kinase (PI-3K), and cyclin-dependent kinases (CDK) that could cooperate with CV-B5/F (DAUC ≥ 0.5). Interestingly, these inhibitors could be involved in a correlative DNA damage response (DDR) pathway. The DNA-PK inhibitor NU7441 and ATM inhibitor KU60019 were identified as the best sensitizers for CV-B5/F.

To evaluate the therapeutic effect, a panel of cell lines were tested for synergistic effects. As shown in Fig. 3b–d and Supplementary Fig. 3b–d, NU7441 sensitizes NCI-H460, A549, PANC-1, and Bxpc3 cells to the oncolytic effects of CV-B5/F. However, NCI-H1299 and Hep3B could not be sensitized by a combination of NU7441. As DNA-PK and ATM are key kinases activated by DNA double-stranded breaks (DSBs), we assessed DSBs in NCI-H460 cells induced by the combination of CV-B5/F and NU7441/KU60019 using the neutral comet assay. NU7441, KU60019, or CV-B5/F treatment alone slightly increased tail moments, whereas the combination of NU7441 or KU60019 with CV-B5/F significantly induced DSBs (Fig. 3e, f). The DSBs marker p-H2AX was demonstrably activated, accompanied by inhibition of DNA-PK or ATM (Fig. 3g and Supplementary Fig. 3e, f). Next, we sought to determine whether the combination induced increased viral replication, apoptosis, and autophagy. As shown in Fig. 3h and Supplementary Fig. 3f, these two combinations induced more cleaved-caspase 3 and VP1 protein. Furthermore, the combination of CV-B5/F and NU7441 promoted autophagy flux (Fig. 3i, j). Another DNA-PK inhibitor, NU7026, also demonstrated evident caspase 3 cleavage by inhibiting DNA-PK (Supplementary Fig. 3g). Eventually, exacerbated infection with CV-B5/F resulted in the release of HMGB1 from the nucleus into the cytosol and supernatant. This can induce immunogenic cell death (ICD)^24^ (Supplementary Fig. 4a). It has been proven that cleaved-caspase 3 priorly cleave gasdermin-E (GSDME) if it was expressed.^25^ We discovered that the enhanced cleaved-caspase 3 promoted GSDME cleavage by the combination of NU7441 and CV-B5/F in NCI-H460 cells (Supplementary Fig. 4b). Collectively, inhibitors of DNA-PK or ATM can synergize with CV-B5/F by exacerbating apoptosis, autophagy, and viral propagation.

### DNA-PKI/ATMI and CV-B5/F cooperate to trigger irreversible ER stress

**Fig. 4:**
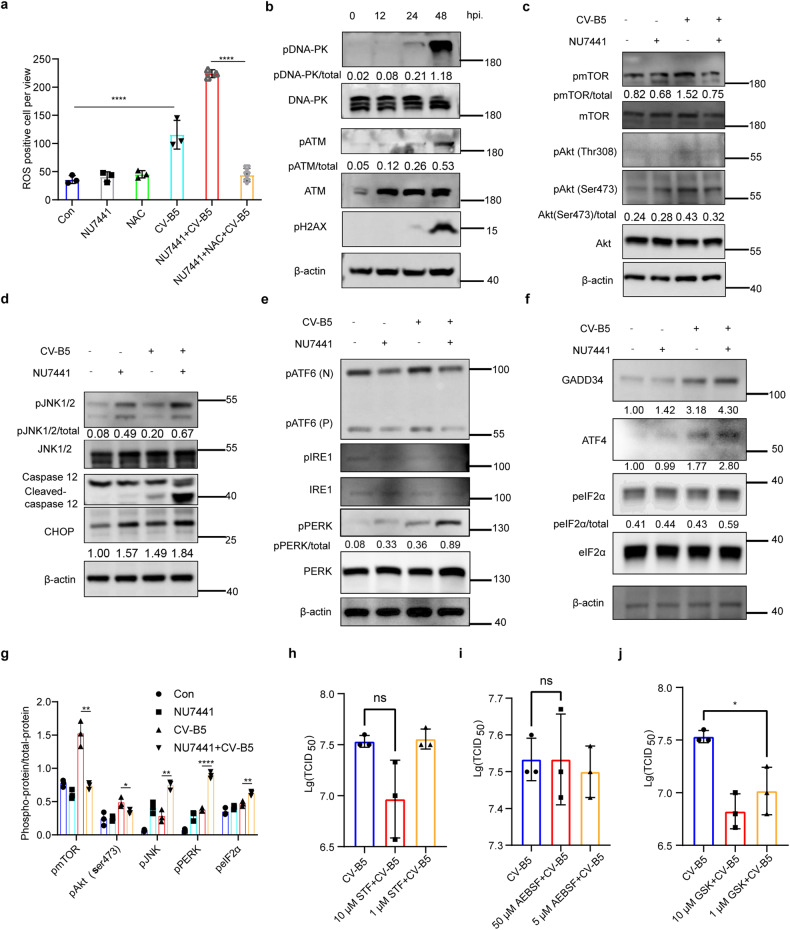
DNA-PK inhibitors plus CV-B5/F virus-induced irresolvable ER stress-associated apoptosis in NCI-H460 cells. **a** NCI-H460 was treated with 1 μM NU7441, 10 μM NAC, 0.01 MOI CV-B5/F, or a combination of NU7441 and CV-B5/F with or without 10 μM NAC. Reactive oxygen species (ROS) of each were detected (*n* = 3). **b** Immunoblots of p-DNA-PK, p-ATM and p-H2AX after infection with CV-B5/F (MOI = 0.01) for 0, 12, 24, and 24 h. **c**–**g** NCI-H460 was treated with 1 μM NU7441, 0.01 MOI CV-B5/F, or a combination for 24 h. Immunoblots of p-mTOR, p-Akt (Ser 473), and p-Akt (Thr 308) were detected (**c)**. ER stress-associated apoptotic pathways, including caspase 12, CHOP (C/EBP-homologous protein), JNK1/2 (Jun N-terminal kinase 1/2) were analyzed by western blot (**d)**. Three initiators of ERS, including ATF6, IRE1, and PERK were detected (**e)**. Immunoblots of downstream of PERK were detected (**f)**. Data of densitometry analysis were shown (**g)**. **h**–**j** NCI-H460 was treated for CV-B5/F (MOI = 1) with or without STF-083010 (an inhibitor for IRE1, **h),** AEBSF (an inhibitor for ATF6, **i)** or GSK2606414 (an inhibitor for PERK, **j)** for 24 h. Viral titers in supernatant were measured (*n* = 3). One-way ANOVA was used to analyze data. *P < 0.05, **P < 0.01, ****P < 0.0001, ns, not significant

RNA viruses can cause a DDR by inducing reactive oxygen species (ROS).^26,27^ We found that CV-B5/F infection can induce ROS release, which can be exacerbated by NU7441 (Fig. 4a). In addition, CV-B5/F infection increased p-DNA-PK, p-ATM, and p-H2AX levels in NCI-H460 cells (Fig. 4b). These results implied that CV-B5/F infection caused DNA damage and activated DNA-PK and ATM, which could be suppressed by NU7441 or KU60019, resulting in extensive DNA damage and cell death. DNA-PK and ATM are members of the PI-3K-related protein kinase.^28^ It can induce Akt, p53, and many other pathways to regulate signal transduction.^29^ Generally, the Akt pathway can adjust the balance of anti-apoptotic (eg. bcl-2, bcl-xl, bad), and pro-apoptotic (eg. bax) molecules, and mTOR phosphorylation affects both the apoptosis and autophagy pathways.^30,31^ Initially, we detected p-Akt and p-mTOR, and the results indicated that Akt and mTOR were activated by viral infection but were inhibited when imposed with NU7441 (Fig. 4c). In addition, we found that the combinatorial use of NU7441 and CV-B5/F caused downregulation of p-bad and the upregulation of bax, which can promote apoptosis (Supplementary Fig. 4c). As a result, the inhibition of DNA-PK can promote apoptosis and autophagy by regulating the Akt pathway.

Viral protein synthesis must be processed in ER.^32^ Abnormal accumulation of misfolded proteins in this compartment causes a state of “ER stress”. Foot and mouth disease viruses can cause ER stress, resulting in ER stress-mediated autophagy.^33^ Hence, we inferred that DNA-PK inhibition enhances prolonged and severe ER stress, eventually inducing cell death. Previously, ER stress-related pro-apoptotic markers, including cleaved-caspase 12, CHOP, and p-JNK, implied that the combination of NU7441 and CV-B5/F induced robust cell death prompted by ER stress (Fig. 4d,g). Furthermore, ER stress markers, including p-IRE1, p-PERK, and ATF6 (N and P), which are the beginning of three activating pathways, were detected.^34^ The results showed that the PERK pathway was activated with robust upregulation of p-PERK (Fig. 4e,g). The downstream protein markers of PERK, including p-eIF2α, ATF4, and GADD34, were also up-regulated (Fig. 4f,g). We also used three inhibitors specific for IRE1, PERK, and ATF6 to treat cells which were then infected with CV-B5/F. Viral titers in the supernatants were measured. The results indicated that only the PERK inhibitor GSK2606414 inhibited viral propagation, which is consistent with the aforementioned results (Fig. 4g–j). These results indicated irreversible ER stress was activated, and it started aggravated cell death.

Similarly, we used siRNA to knock down DNA-PK and ATM and then infected cell with CV-B5/F. We found that the expression of p-H2AX, cleaved-caspase 3, and lipidated LC3B (LC3B II) was upregulated in siDNA-PK-treated and siATM-treated groups compared to the negative control group after CV-B5/F infection. In addition, ER stress markers such as PERK, p-eIF2α and ATF4 were upregulated, indicating that knock-down of DNA-PK or ATM in NCI-H460 cells aggravated ER stress-associated cell death (Supplementary Fig. 5a–f). Thus, the knockdown of DNA-PK or ATM also showed similar effect as inhibitors.

To validate the relationship of ROS and PERK pathway, we used the ROS scavenger α-NAC and GSK2606414 to pretreat NCI-H460 cells. The results indicated that ROS release could be inhibited by the PERK inhibitor GSK2606414 (Supplementary Fig. 5g, h). Collectively, ROS was also important for the activation of PERK pathway.

### DNA-PKI or ATMI plus CV-B5/F was effective in vivo

**Fig. 5:**
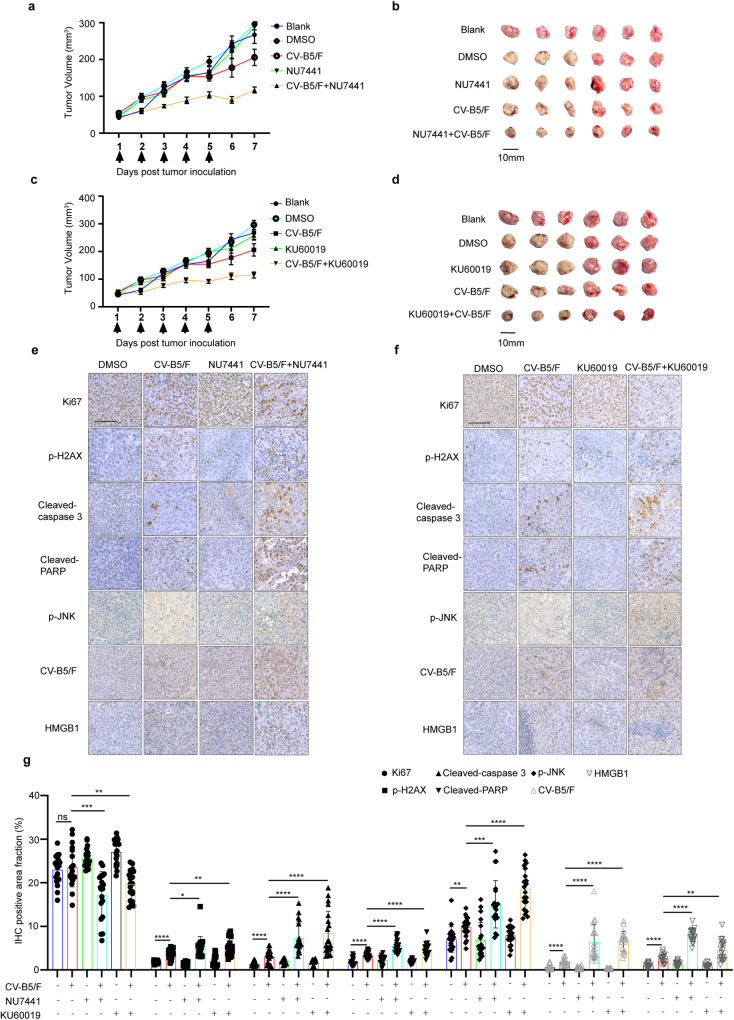
Inhibiting DNA-PK or ATM enhances oncolytic therapy in BALB/c nude models of NCI-H460 xenografts. **a**, **b** NCI-H460 xenografts were treated with vehicle, CV-B5/F (5 × 10^6^ TCID_50_, intratumorally injection), NU7441 (10 mg/kg/day, intraperitoneally) or a combination. Blank, *n* = 5; Vehicle, *n* = 5; CV-B5/F, *n* = 5; NU7441, *n* = 5; NU7441 + CV-B5/F, *n* = 5. Tumor growth presented as the mean tumor volume ± SEM (**a)**. Images of tumors from each group in (**a**) at the experimental endpoints (**b)**. **c,** **d** NCI-H460 xenografts were treated with vehicle, CV-B5/F (5 × 10^6^ TCID50, intratumorally injection), KU60019 (10 mg/kg/day, intraperitoneally) or a combination. Blank, *n* = 5; Vehicle, *n* = 5; CV-B5/F, *n* = 5; KU60019, *n* = 5; KU60019 + CV-B5/F, *n* = 5. Tumor growth presented as the mean tumor volume ± SEM (**c)**. Images of tumors from each group in (**c**) at the experimental endpoints (**d)**. **e**–**g** Tumor tissues from B (**e)** or D (**f)** were evaluated through IHC for Ki-67 (a marker of proliferation), p-H2AX, cleaved-caspase-3, cleaved-PARP, p-JNK, CV-B5/F, HMGB1. One-way ANOVA was used to analyze data (*n* = 20). *P < 0.05, **P < 0.01, ***P < 0.001, ****P < 0.0001, Scale bar, 100 μm

To evaluate the anticancer activity of CV-B5/DNA-PKI and CV-B5/ATMI in vivo, mice bearing subcutaneous NCI-H460 CDX were treated with (i) intratumoral CV-B5/F, (ii) intraperitoneal NU7441/KU60019 injection, or (iii) a combination of i and ii. CV-B5/F plus NU7441/KU60019 treatment restricted tumor growth compared with monotherapy alone (Fig. 5a–d). At the endpoint, the levels of Ki67, p-H2AX, cleaved-caspase 3, cleaved-PARP, p-JNK, CV-B5/F, and HMGB1 were further examined in subcutaneous xenograft tumor sections by IHC. We observed that Ki67 levels were relatively low in the combinatorial treatment group, indicating that the proliferation of tumor cells was inhibited by CV-B5/F plus NU7441 or KU60019. Furthermore, p-H2AX, cleaved caspase 3, cleaved-PARP, p-JNK, CV-B5/F, and HMGB1 were elevated in the combinatorial treatment group compared with the single treatment groups (Fig. 5e–g). The results showed that combination therapy elicited severe DNA damage, increased viral propagation, and insurmountable ER stress-related apoptosis and ICD.

### ER stress induces ERphagy-related STING degradation to promote viral replication

**Fig. 6:**
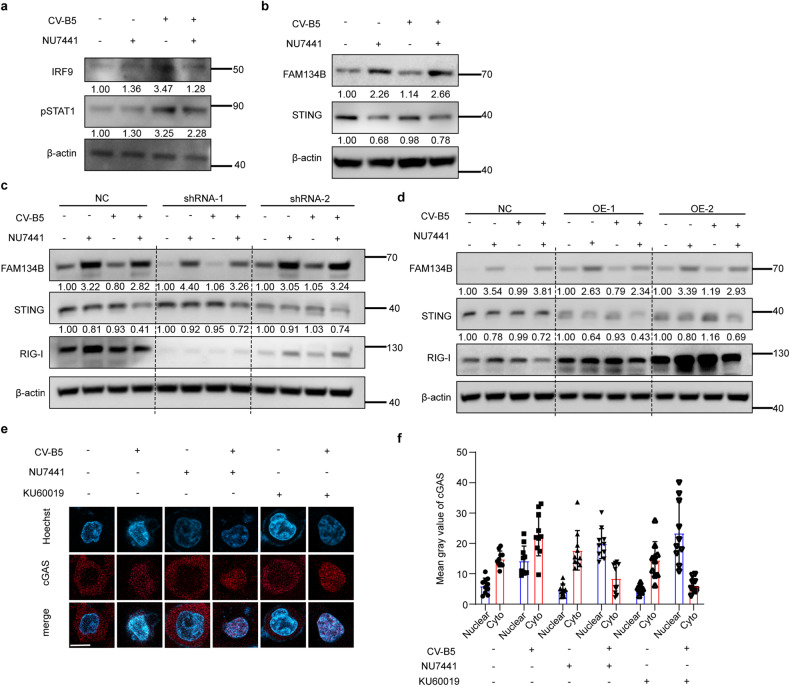
Exacerbated ERphagy induced by the combination of NU7441 and CV-B5/F degraded STING activated by nuclear cGAS recruited by DSBs. **a** NCI-H460 was treated with 1 μM NU7441, 0.01 MOI CV-B5/F, or a combination for 24 h. Immunoblots of anti-viral proteins including IRF9 and p-STAT1 were detected. **b** NCI-H460 cells were treated with 1 μM NU7441, 0.01 MOI CV-B5/F, or a combination for 24 h. FAM134B (a marker of ERphagy) and STING were determined by western blot. **c** NCI-H460 cells knocked down with shRNA for RIG-I were treated with 1 μM NU7441, 0.01 MOI CV-B5/F, or a combination for 24 h. RIG-I, FAM134B and STING were determined by western blot. Gray values of NC, shRNA-1 and shRNA-2 were compared separately. **d** NCI-H460 cells overexpressing RIG-I were treated with 1 μM NU7441, 0.01 MOI CV-B5/F, or a combination for 24 h. RIG-I, FAM134B and STING were determined by western blot. Gray values of NC, OE-1 and OE-2 were compared separately. **e**, **f** NCI-H460 cells were treated with 1 μM NU7441 or 1 μM KU60019, 0.01 MOI CV-B5/F, or a combination for 24 h. Immunofluorescence of cGAS of nuclear was analyzed by confocal microscopy. Mean gray value of cGAS in nuclear and cytoplasma was separately analyzed by Image J software (*n* = 20)

To understand the mechanism of upgrading viral propagation when using these inhibitors, we first detected some pivotal markers that regulate anti-viral responses, including interferon Regulatory Factor 9 (IRF9) and phosphorylated signal transducer and activator of transcription 1 (p-STAT1).^35^ The results indicated that viral infection activated the anti-viral response, manifesting as the upregulation of IRF9 and p-STAT1. However, amalgamation of NU7441 reversed this tendency (Fig. 6a). STING, located in the ER, is a crux that can be activated during viral infection.^36,37^ The expression of STING can be affected when ER-associated autophagy (ERphagy) is activated. As a result, we studied STING and the ERphagy marker-FAM134B to elucidate this speculation. As shown in Supplementary Fig. 6a, b, STING was activated by CV-B5/F which can be inhibited when using NU7441, and the activation of IRF3 was also inhibited meanwhile. Remarkably, STING was degraded because of the upregulation of FAM134B (a marker of ERphagy) treated with NU7441 (Fig. 6b), because infection of CV-B5/F could not upregulate FAM134B, and treatment with NU7441 can cause ERphagy conversely. These results indicated that STING was degraded by ERphagy.

The cGAS-STING pathway that recognizes DNA virus infection is canonical when it comes to STING activation.^38^ However, as an RNA virus, infection with CV-B5 stimulates cGAS and often activates RIG-I, which is another activator of STING.^37,39^ By knocking down or overexpress RIG-I in NCI-H460 cell, we also found similar phenomenon by contrast to control (Fig. 6c, d, Supplementary Fig 6c, d). To further elucidate the degradation of ER, we used a plasmid encoding ER retention sequence (KDEL-GFP) to transfect NCI-H460 cell. KDEL-GFP also showed downregulation in the meantime with STING, and it can be inhibited by CQ (Supplementary Fig 6e). Moreover, wild-type HEK293, *RIG-I*-stable overexpression (OE) HEK293, and *RIG-I*-KO HEK293 cells were used to compare the activation of the STING pathway. It is important to note that ER stress could be induced by CV-B5/F infection in these three cell lines. As shown in Supplementary Fig. 7a, ER stress was activated in three cell lines infected with CV-B5/F and NU7441. Furthermore, NU7441 promoted viral replication by suppressing DNA-PK activation (Supplementary Fig. 7b). However, NU7441 also enhanced the level of FAM134B in these three cell lines and promoted STING degradation even in *RIG-I* OE cells (Supplementary Fig. 7c). A similar phenomenon was observed in KU60019 pretreated cells (Supplementary Fig. 7d). In summary, RIG-I was not the upstream of STING in this study.

When DNA damage is severe, broken DNA can recruit cGAS from the cytoplasm into the nucleus, activate it, and then induce cGAMP, which is a second messenger that activates STING or epigenetic modifications of the promoters of type I IFNs.^40,41^ In this study, we found that the combination of NU7441 and CV-B5/F exacerbated DNA damage and further recruited cGAS into the nucleus to activate STING. As shown in Supplementary Fig. 6f, cGAS in the cytoplasm was reduced after treatment with NU7441 or KU60019 and CV-B5/F, while cGAS in the nucleus was increased. Immunofluorescence staining for cGAS indicated that the cGAS co-located with nuclear increased after the combination treatment of NU7441 or KU60019 with CV-B5/F (Fig. 6e, f). Together, NU7441 and KU60019 could simultaneously exacerbate DNA damage and ERphagy at the same time. Broken DNA can recruit cGAS into the nucleus and activate the anti-viral response, which is a restraining factor for oncolytic viruses. Meanwhile, the ERphagy caused STING degradation, which was located on the ER membrane, which reversed this phenomenon.

## Discussion

**Fig. 7:**
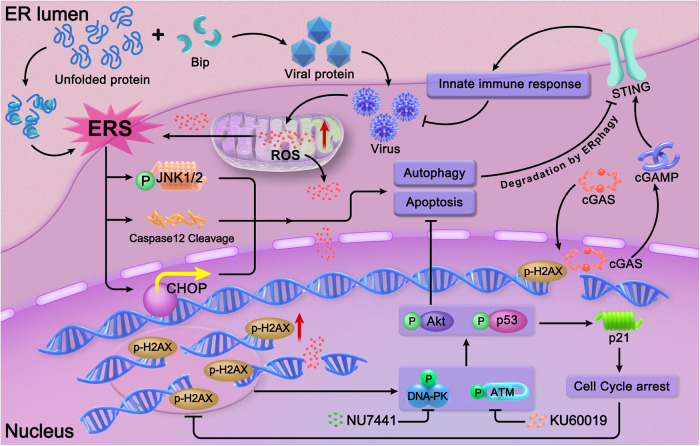
Graphical model of DNA-PK or ATM and CV-B5/F combination therapy. Normally, viral proteins are folded in the ER and assembled in the cytoplasm. Massive accumulation of unfolded viral proteins can induce ERS, which is initiated by the combination of bip and unfolded proteins. Irresolvable ERS can induce apoptosis, and autophagy which relies on the activation of p-JNK, caspase 12, and CHOP. In addition to inducing ERS, viral propagation can also induce the release of ROS from the mitochondria, which can cause DSBs. DSBs can further activate the DNA damage response, including the activation of Akt, which inhibits apoptosis and autophagy, and the activation of p53, which causes cell cycle arrest and diminishes DSBs. Thus, co-treatment with DNA-PKI or ATMI hinders DNA repair and promotes DNA damage induced by CV-B5/F. Furthermore, excess DSBs can recruit cGAS into the nucleus and activate the STING pathway for the innate immune response. However, ERphagy, a form of autophagy, is also initiated by inhibitors and can degrade STING to offset this response. Above all, DNA-PK or ATM inhibitors can synergize with CV-B5/F by attenuating the antiviral pathway and increasing DNA damage-mediated cell apoptosis and autophagy

Oncolytic enteroviruses, including CV-A21, CV-B3, Echovirus 7, have been reported to have robust anti-tumor abilities. However, the high seroprevalence of multiple enteroviruses necessitates the development of other oncolytic enteroviruses as promising drugs for cancer treatment. In this study, we reported that CV-B5/F had potent anti-tumor activity against NSCLCs by inducing apoptosis and autophagy. For refractory NSCLC cells, such as NCI-H460, DDR inhibitors can synergize with CV-B5/F by exacerbating ER stress-related cell death and viral propagation to develop oncolytic effects (Fig. 7).

CV-B5 is the pathogen that induces HFMD, a few of which progress to aseptic encephalitis only in infants and children.^42^ It relies on specific receptors to enter the cell membrane, including the main receptor-CAR and co-receptor-DAF. Although it shows tissue tropism for neurons, the receptor expression level in mature neurons becomes less susceptible to CV-B5 because of the decrease in CAR levels compared with immature neurons.^43^ Wang et al. also indicated that CAR is specifically expressed in various histological types of human lung cancers, but not in normal alveolar epithelial cells.^44^ In our study, compared to universal and homogeneous expression of DAF, CAR expression showed specificity in lung cancer cells with its oncolytic effect. To further demonstrate the significance of CAR, we overexpressed CAR in LLC and CT26.WT cells, and the expression level of CAR correlated with cell viability. These data indicate that CV-B5/F can selectively lyse CAR-expressing cancer cells, ensuring specificity and security.

After entering host cells, OV can employ the copy and translational system to complete the infection cycle, interfere with host cell metabolism, and eventually kill cells.^45^ Normally, the balance and coordination between pro-apoptotic and anti-apoptotic factors can eliminate abnormal cells and maintain an equilibrium.^46^ Overexpression of anti-apoptotic proteins occurs in most tumor cells, in which apoptosis is suppressed. Many OVs can reverse this phenomenon. For instance, CV-B3 can activate the apoptotic pathway to mediate the death of lung cancer.^9^ In this study, after administration of CV-B5/F, apoptosis and autophagy were activated not only in multiple NSCLC cells but also in an immunocompetent mouse model, indicating that apoptosis is one of the pathways responsible for cell death.

In this study, a series of animal models were used to evaluate oncolytic effects. For the immunocompetent BALB/c nude mice model, it is noteworthy that intratumoral CV-B5/F administration showed a significant anti-tumor effect against A549 and NCI-H1299 xenografts but showed no anti-tumor effect against NCI-H460 xenografts. In particular, the long-term observation of NCI-H1299 xenografts for 60 days indicated that no rebound occurred. Intratumoral administration into one of the bilateral subcutaneous xenografts elicited significant suppression of growth of distant uninjected tumors, which implies that propagated CV-B5/F can circulate via the blood or lymphatic system and target distant tumors, which is beneficial for the systemic treatment of metastatic or disseminated tumors.

Apart from the direct oncolytic effect, many OVs can induce systemic anti-tumor responses, manifesting as specific T cells being recruited and infiltrating.^47^ In the LLC-CAR model of immunocompetent mice, CV-B5/F induced significant CD4^+^ and CD8^+^ T-cell infiltration. However, there was a limitation in that they were composed of both murine immune and tumor cells. Therefore, we constructed a humanized mouse model based on *β2m* knockout B-NDG and human PBMCs, which can prolong the observation window period by eliminating MHC class I-related delayed development of graft versus host disease.^22^ Administration of CV-B5/F can induce apoptosis and autophagy and recruit eminent T cell infiltration.

The PDX model relies on direct transplantation of tumor specimens obtained during surgery or from biopsies into an immunocompromised mouse, providing a faithful representation of individual tumors compared to the CDX model.^48^ In this study, we constructed a PDX model by transplanting patient-derived NSCLC tumors. Five injections of CV-B5/F inhibited (5/5) tumor growth, which confirmed the oncolytic effect of CV-B5/F.

To ensure safety, normal lung cell lines were infected with a high MOI, and these cells did not show any despair. For in vivo safety, BALB/c nude mice inoculated with five injections of CV-B5/F were used for histopathological analysis, the results of which also confirmed the safety.

To elucidate the problem and cope with refractory tumors, such as NCI-H460, we performed a large-scale screening of drugs with synergistic therapeutic effects. As a result, a series of inhibitors against the DDR pathway and its downstream molecules can synergize with CV-B5/F to some extent, which means that the pathway can be activated by viral infection and protect tumor cells from death. We observed that ROS induced by CV-B5/F infection caused DDR, which repaired the DSBs and promoted cell survival. The results showed that DNA-PKI or ATMI revert DDR and induce ER stress-related cell death.

However, some tumor cells, including NCI-H1299 and Hep3B, which are sensitive to CV-B5/F and p53 deleted, showed no difference after NU7441 treatment. This prompted us to study the characteristics of the p53 pathway. In general, p53 is activated during DSB occurrence. Specifically, p-DNA-PK or p-ATM can phosphorylate ChK1 or ChK2, and p-ChK1 and p-ChK2 can phosphorylate p53 and promote the phosphorylation of p53 at Ser 15 and Ser 37. Phosphorylated p53 can upregulate p21, which is the main factor that arrests the cell cycle and promotes DNA damage repair. If DNA damage is too severe to repair, these cells can initiate death by upregulating PUMA and NOXA.^49^ In the present study, CV-B5/F infection significantly upregulated the expression of p-p53 (Ser 15) and p21. Nevertheless, the combination of NU7441 and ATM reduced p-p53 and p21 (Supplementary Fig. 8a, b). Thus, NU7441 and KU60019 can repress the activation of the p53 pathway to avoid cell cycle arrest (Supplementary Fig. 8c). Oncolytic effect of CV-B5/F on NCI-H1299 overexpressed with p53-WT or p53-R273H mutant can be synergized by NU7441 or KU60019 manifesting as exacerbated caspase 3 cleavage and H2AX phosphorylation (Supplementary Fig. 8d–f). As a result, DNA-PKI or ATMI cannot synergize with CV-B5/F in p53-deficient tumor cells.

Another result we obtained that was contrary to our expectation was that DNA-PK or ATM inhibitors could promote CV-B5/F propagation by restraining the anti-viral effect (Fig. 5a). STING is located at the ER membrane upstream of IFN. We observed that STING was activated by viral infection and can be inhibited by the combinatorial treatment of NU7441 and KU60019. STING is canonically activated by cGAS in DNA viruses or RIG-I in RNA viruses.^37–39^ Nonetheless, STING activation was detected in *RIG-I* KO HEK293 cells. Simultaneously, *RIG-I* OE exhibited STING degradation induced by DNA-PK or ATM inhibitors. As a result, RIG-I did not appear to be upstream of STING in our study. Cui et al. found that cGAS can be recruited to the nucleus by broken DNA and activated to induce cGAMP.^41^ In this study, we found that aggravated DSBs induced by viral infection recruited cGAS to the nucleus. In contrast, DNA-PK or ATM inhibitors plus CV-B5/F can induce ERphagy to degrade STING, which diminishes the anti-viral effects caused by the overactivated cGAS.

For enterovirus group B, coxsackievirus B3 Nancy strain showed an oncolytic activity in xenografted mouse models of NSCLC, small cell lung cancer, *KRAS*-mutant lung adenocarcinomas, and colorectal carcinomas by specific receptors on cancer cell, including CAR, DAF, and heparan sulfates (HS). However, to my certain knowledge, those oncolytic effects were only studied in CDX models, and some studies observed moderate virus-induced diseases, including pancreatitis, hepatic dysfunction and mild myocarditis.^50^ In contrast, CV-B5/F demonstrated an impressive oncolytic effect in NSCLCs not only in CDX but also in PDX model, and no treatment-related death or diseases were observed.

Targeting non-homologous end joining (NHEJ) and homologous recombination (HR) as a therapeutic intervention in human tumor, especially to sensitize tumor cells to chemotherapy or radiotherapy, has recently been seemed to showed great clinical prospect. For example, using NU7441 to knockdown DNA-PK in NHEJ enhanced the sensitivity to radiation therapy in colon, breast and ovarian cancers.^51–54^ Compared to the NHEJ repair pathway, HR-mediated DSB repair pathway is tended to be employed occurring DSBs. Thus, understand relative mechanisms could benefit the development of novel approaches for cancer therapy. For example, AZD0156, a ATM inhibitor, can enhance the breast tumor growth inhibitory effects of radiation treatment in vivo.^55^ Furthermore, inhibitor targeting NHEJ was also reported for a oncolytic M1 virus, which showed great synergistic effects.^56^ However, other drugs targeting DDR to enhance the therapeutic effect of other OVs still lack.

In summary, our study identified CV-B5/F as a safe and tumor-specific oncolytic virus for NSCLCs, which has been validated in multiple CDX and PDX mouse models. Furthermore, DDR inhibitors can provide a significant therapeutic benefit in combination with CV-B5/F for the treatment of refractory cancers by exacerbating viral replication, DSBs, and multiple pathways of cell death.

## Materials and methods

### Mice

Four-week-old female BALB/c nude mice and BALB/c and C57BL/6 mice were purchased from NIFDC. B-NDG mice were purchased from Biocytogen Pharmaceuticals Co. Ltd. (Beijing, China). NPI (NOD-SCID, *Fah*^−/−^, *Il12rg*^−/−^) mice were purchased from the IMDO. All animal research protocols were approved by the Institutional Animal Care and Use Committee at National Institutes for Food and Drug Control, China (No. 2022-B002), and these protocols were conducted in accordance with the regulations on the management of laboratory animals (National Science and Technology Commission no. 2 of Oct. 31, 1988) and “guidance notes on the treatment of experimental animals” (Chinese version (2006) no. 398). All institutional guidelines for animal care and use were strictly followed throughout the experiments.

### Cell lines

NSCLC (A549, NCI-H1299, NCI-H460), liver cancer (HepG2, PLC/PRF/5, Hep3B), cervical cancer (HeLa), rhesus monkey kidney (LLC-MK2), African green monkey kidney (Vero), rhabdomyosarcoma (RD), and human normal lung fibroblast (MRC-5, WI-38, and HFL) cell lines were preserved in the National Institutes for Food and Drug Control. Mouse Lewis lung carcinoma (LLC) and mouse colorectal carcinoma (CT26.WT) cells were purchased from the American Type Culture Collection (ATCC). Lucia HEK293 cells and Lucia HEK293 cells expressing human RIG-I were purchased from InvivoGen. NCI-H1299 cells stably expressing firefly luciferase (NCI-H1299-Luc) were transfected with pcDNA3.1-Luc plasmid using Lipo3000 (Thermofisher, L3000150), and the single clone was screened by G418 (Thermo Fisher, 10131035). LLC and CT26.WT cells stably expressing coxsackievirus and adenovirus receptor (CAR) and firefly luciferase (LLC-CAR-Luc and CT26.WT-CAR-Luc) were generated by fusion expression by lentivirus transfection.

### Viruses

The prototype and isolated strains of CV-B3, CV-B5, and CV-A6 were used in this study. The prototype strain CV-B3/Nancy (GenBank: M88483), CV-B5/Faulkner (CV-B5/F, GenBank: AF114383), and CV-A6/Gdula (GenBank: AY421764) were purchased from ATCC and propagated in Vero, LLC-MK2, and RD media, respectively. CV-B5/JS417^57^ (GenBank: KY303900) and CV-B3/112^58^ (GenBank: KP036480) strains were isolated from a human throat swab sample of a 2-year-old boy and a 2-year-old girl, respectively, were obtained from a previously described clinical hand foot and mouth disease case collection at Jiangsu Province Center for Disease Control and Prevention and was anonymized in mainland China in 2013, and it were propagated in LLC-MK2 and Vero respectively. The TCID_50_/mL on these cell monolayers was determined as previously described.^59^ Virus information is presented in Supplementary Table 1.

### Reagents and inhibitors

Z-VAD-FMK, chloroquine (CQ), Rapamycin (Rapa), NU7441, Cyt387, A674563, KU60019, LY294002, PD0332991, AT7519, MK2206, CUDC907, LY2109761, GANT61, BIX 02189, Spautin-1, QNZ, LY317615, PD169316, GSK2606414, LYK974, TCK ERK 11e, SC79, MC1568, H89, ICG001, Perifosine, AEE788, ABT263, GDC-0941, FH535, PD0325025, NU7026, STF-083010, AEBSF HCl were purchased from Adooq. N-Acetyl-l-cysteine ethyl ester, DMSO, PEG300, and Tween-80 were purchased from MCE.

### Cell viability assay

Cells were seeded in 96-well plates at 10000 cells/well in 0.1 mL of medium. After treatment, CCK8 reagent (Dojindo, CK08) was added to the cells at 11 μL per well and the cells were allowed to grow at 37 °C. The optical density (OD) was determined at 450 nm using a microplate reader (X MARK, Bio-Rad) when the OD value of the control well reached 1.0 approximately.

### In vivo therapeutic studies in BALB/c nude, BALB/c, C57BL/6, and B-NDG mice

For xenografts in BALB/c nude mice, 5 × 10^6^ cells (A549, NCI-H1299, NCI-H460) were injected subcutaneously into the right or bilateral flanks of BALB/c nude mice. For LLC and CT26.WT cells, 5 × 10^5^ cells were implanted into the axillia of C57BL/6 and BALB/c mice, respectively. When tumors reached diameters of 4–6 mm or 7–8 mm, the tumors were inoculated with CV-B5/F (5 × 10^6^ TCID_50_) or with other inhibitors dissolved in 10% DMSO + 40% PEG300 + 5% Tween-80 + 45% saline intratumorally for different procedures. Tumor length and width were measured every day or every two days.

For luciferase-expressing NCI-H1299, LLC, and CT26.WT, mice were intraperitoneally administered the bioluminescent substrate D-luciferin (75 mg/kg, 15 mg/mL, PerkinElmer) before imaging and then anesthetized with pentobarbital sodium intraperitoneally (75 mg/kg, 15 mg/mL). After 10 min, the bioluminescence intensity of the mice was measured using an IVIS-Lumina II imaging system (Xenogen). The relative intensities of the bio-optical signals were coded from red (intense) to blue (weak) and presented quantitatively as photon flux in log average radiance (p/s/cm/sr).^60^

For B-NDG mouse model, seven days post-tumor injection, peripheral blood mononuclear cells (PBMC, Oribiotech, #PB004-C) were thawed in a 37 °C water bath, followed by three washes with RPMI containing 10% FBS and one wash with PBS. PBMCs (1 × 10^7^) were injected intraperitoneally (i.p.) in 200 µL PBS. 5 × 10^6^ (NCI-H1299-Luc) cells were combined with Matrigel at a 1:1 ratio immediately prior to subcutaneous (s.c.) injection into the axillia of recipient animals in a total volume of 100 µL. Other procedures were identical to those described previously.^22^

When the diameters were more than 15 mm, mice were considered to be reaching the existing terminal ethically. Tumor volume was calculated using the following formula: (length×width^2^)/2. After anesthetization, samples were collected from the brain, heart, lungs, liver, spleen, kidneys, pancreas, and tumors.

### In vivo patient-derived xenograft model

Primary tumor tissue specimens were obtained from the patients who underwent tumor resection. Tumor samples were collected in a cell culture medium and processed within 4 h. Samples were manually divided into ~1 mm^3^ blocks using a scalpel blade under sterile conditions. The explants were placed on moist but not completely submerged filter paper inserted into each well of 24-well plates with 1 mL DMEM containing 15% (vol/vol) FBS until the explants were inoculated subcutaneously into hind-flanks of 4-week-old male NPI mice mouse within 4 h. After 4–6 weeks, palpable tumors developed ( ~ 50 mm^3^) and the mice were randomly divided into two groups. CV-B5/F (5 × 10^6^ TCID_50_/day) and MEM were administered by intratumoral injection to the CV-B5/F-treated and untreated groups, respectively. Tumor length and width were measured daily, and the volume was calculated according to the formula (length×width^2^)/2. When the diameter reached 10 mm, it could be considered as ethically reaching the existing terminal. After anesthetization, the tumor samples were dissected. The study was approved by the ethics review committee of the Peking University People’s Hospital (Beijing, China).

### Hematoxylin and eosin staining for animal tissues

After anesthetization, samples were collected from the brain, heart, lungs, liver, spleen, kidneys, and pancreas. The mouse tissues were fixed by immersion in 10% neutral buffered formalin for at least 2 days. The tissues were bisected and embedded in paraffin. Tissue sections were stained with hematoxylin and eosin.

### Immunohistochemistry assay

The expression of Ki67, cleaved-caspase 3, CD3, CD4, CD8, granzyme B, cleaved-PARP, p-JNK, p-H2AX, CV-B5/F, and HMGB1 in the tumors was assessed by immunohistochemistry. Briefly, tumor sections (4 μm) were dewaxed in xylene, hydrated in decreasing concentrations of ethanol, immersed in 0.3% H_2_O_2_-methanol for 30 min, washed with phosphate-buffered saline, and probed with antibodies at 4°C overnight. After washing, sections were incubated with biotinylated goat anti-rabbit or anti-mouse IgG at room temperature for 2 h. Immunostaining was visualized with streptavidin/peroxidase complex and diaminobenzidine, and sections were counterstained with hematoxylin. The following antibodies were used: Ki67 (1:500, 9027, Cell Signaling Technology), cleaved caspase 3 (1:2000, 9664, Cell Signaling Technology), CD3 (1:200, 85061, Cell Signaling Technology), CD4 (1:500, 133616, Abcam), CD8 (1:200, 85336, Cell Signaling Technology), granzyme B (1:100, 46890, Cell Signaling Technology), cleaved PARP (1:50, 5625, Cell Signaling Technology), p-JNK (1:50, 4668, Cell Signaling Technology), p-H2AX (1:50, 4668, Cell Signaling Technology), CV-B5/F (1:2000, self-prepared), and HMGB1 (1:200, 6893, Cell Signaling Technology).

### RNA interference

Specific and scramble siRNAs were purchased from Ribobio. shRNA were purchased from MIAO LING BIO. siRNAs were transfected using Lipofectamine RNAiMAX (13778-150, Thermo Fisher Scientific) with OPTI-MEM (31985070, Thermo Fisher Scientific). shRNAs were transfected using Lipofectamine 3000 (L3000015, Thermo Fisher Scientific) with OPTI-MEM (31985070, Thermo Fisher Scientific).

### Flow cytometry analysis

For receptor detection, cells were incubated with a PE-conjugated monoclonal antibody against CAR (sc-56892, Santa Cruz Biotechnology) and normal mouse antibody (sc-2866, Santa Cruz Biotechnology), followed by incubation with FITC-conjugated monoclonal antibody against CD55 (sc-51733, Santa Cruz Biotechnology).

For the detection of tumor-infiltrated lymphocytes, fresh tumors were digested with collagenase (17104019, Thermo Fisher) and separated by lymphocyte separation medium (7211011, DAKEWE). Lymphocytes were stained with CD3 (553061, BD Biosciences), CD4 (553051, BD Biosciences), and CD8 (558207, BD Biosciences).

For annexin V staining, cells after CV-B5/F infection were determined using the Annexin V–PE apoptosis detection kit (BD Biosciences) according to the manufacturer’s instructions.

Data were obtained using a FACS Calibur (BD Biosciences) and analyzed using FlowJo software version 7.6.

### Western Blot

Protein lysates (30–50 μg per sample) were separated on 10% or 4–20% SDS-polyacrylamide gels and transferred to NC membranes. Incubation with primary and secondary antibodies and signal detection were performed according to the manufacturer’s protocol using ECL (Thermo Fisher). Antibodies to the following proteins were used in this study: β-actin (1: 1000, 4970, Cell Signaling Technology), GAPDH (1: 5000, ab8245, Abcam), DAF (1: 1000, 31759, Cell Signaling Technology), CAR (1: 1000, 16984, Cell Signaling Technology), DAF (1: 2000, sc-51733, Santa Cruz Biotechnology), PARP (1: 1000, 9532, Cell Signaling Technology), Caspase 3 (1: 2000, 9662, Cell Signaling Technology), Cleaved-caspase 3 (1: 2000, 9664, Cell Signaling Technology), LC3B (1: 2000, L7543, Sigma), p62 (1: 2000, ab109012, Abcam), DNA-PK (1: 1000, 38186, Cell Signaling Technology), p-DNA-PK (1: 1000, 68716, Cell Signaling Technology), ATM (1: 1000, 2873, Cell Signaling Technology), p-ATM (1: 1000, ab81292, Abcam), H2AX (1: 2000, ab229914, Abcam), p-H2AX (1: 1000, 80312, Cell Signaling Technology), Akt (1: 1000, 4685, Cell Signaling Technology), p-Akt (Ser473, 1: 1000, 4060, Cell Signaling Technology), p-Akt (Thr308, 1: 1000, 13038, Cell Signaling Technology), mTOR (1: 1000, 2983, Cell Signaling Technology), p-mTOR (1: 1000, 5536, Cell Signaling Technology), α/β-tubulin (1: 1000, 2148, Cell Signaling Technology), Bad (1: 1000, 9292, Cell Signaling Technology), p-Bad (Ser136, 1: 1000, 4366, Cell Signaling Technology), p-Bad (Ser112,1: 1000, 5284, Cell Signaling Technology), Bax (1: 1000, 5023, Cell Signaling Technology), Bcl-2 (1: 1000, 3498, Cell Signaling Technology), Bcl-xL (1: 1000, 2764, Cell Signaling Technology), AIF (1: 1000, 5318, Cell Signaling Technology), JNK (1: 1000, 9252, Cell Signaling Technology), p-JNK (1: 1000, 4668, Cell Signaling Technology), Caspase 12 (1: 2000, ab62484, Abcam), CHOP (1: 1000, ab11419, Abcam), PERK (1: 1000, 5683, Cell Signaling Technology), p-PERK (1: 1000, 3179, Cell Signaling Technology), IRE1 (1: 2000, ab96481, Abcam), p-IRE1 (1: 2000, ab48187, Abcam), ATF6 (1: 2000, ab227830, Abcam), eIF2α (1: 2000, 5324, Cell Signaling Technology), p-eIF2α (1: 2000, 3398, Cell Signaling Technology), ATF4 (1: 2000, ab184909, Abcam), GADD34 (1: 1000, 41222, Cell Signaling Technology), IRF9 (1: 1000, 76684, Cell Signaling Technology), p-STAT1 (1: 1000, 9167, Cell Signaling Technology), STING (1: 1000, 13647, Cell Signaling Technology), FAM134B (1: 1000, 83414, Cell Signaling Technology), cGAS (1: 1000, 79978, Cell Signaling Technology), Lamin B1 (1: 1000, ab194109, Abcam), HMGB1 (1: 1000, 6893, Cell Signaling Technology), GSDME (1: 1000, 19453, Cell Signaling Technology), Bip (1: 1000, 3177, Cell Signaling Technology), p53 (1: 2000, ab26, Abcam), p-p53 (Ser15, 1: 2000, ab223868, Abcam), p21 (1: 1000, 2947, Cell Signaling Technology), RIG-I (1:1000, 3743, Cell Signaling Technology), STING (1:500, A3575, Abclonal), V5 (1:1000, 13202, Cell Signaling Technology), GFP (1:1000, ab1218, Abcam). Anti-CV-B5 polyclonal antibody (1: 1000) was preserved in our lab.

### Co-immunoprecipitation (Co-IP)

NCI-H460 lysates were extracted using a Pierce Co-Immunoprecipitation Kit (Thermo) according to the manufacturer’s instructions. Briefly, cell lysates were subjected to immunoprecipitation using anti-GFP antibody (ab290, Abcam). The precipitated protein complex was separated and detected by Western Blot assay.

### In vitro apoptosis inhibition assay

Cells were pretreated with serum-free medium containing 100 μM z-VAD-FMK (Adooq) and then exposed to CV-B5/F for an additional 48 h.

### Fluorescent confocal microscopy for autophagy

Semiconfluent (70–80%) monolayers of stably transfected mcherry-GFP-LC3B^61^ (Addgene #110060) cells on glass slides (Cellvis, D.35-14.1-N) were incubated in different media for 48 h. cells were washed with PBS and fixed in 4% paraformaldehyde (Sigma, P6148) at the room temperature for 30 min. After another 3× PBS wash, the samples were stained with Hoechst 33342 (Thermo). Images were acquired using a Leica LSM900^®^ confocal laser-scanning microscope.

### Neutral Comet assay

Cells were seeded in 6-well plates and exposed to different media. Cells were harvested by trypsinization, washed, and resuspended in PBS. DNA double-strand breaks were measured and quantified by single-cell gel electrophoresis using the CometAssay Kit (ab238544, Abcam). Comets were analyzed using the CaspLab software to obtain the average tail moment (TM). At least 20 cells were counted in each sample. Data were analyzed using the Kruskal-Wallis test.

### Nuclear/cytoplasmic fractionation

Nuclear/cytoplasmic fractionation was performed according to Abbkine subcellular and nuclear fractionation protocols. Nuclei were isolated by centrifugation, and the supernatant containing the cytosolic fraction was collected. Equal volumes of nuclear and cytoplasmic lysates were assayed using immunoblotting.

### ROS detection

Cells grown on a 96-well CellCarrier (6055300, PerkinElmer, 1 × 10^4^ cells/well) were treated with different media for 24 h. For ROS detection, cells were processed according to the manufacturer’s protocol (ab113851, Abcam). Cells were imaged with a filter set appropriate for fluorescein isothiocyanate (FITC) using a Perkin Elmer Operetta CLS High Content System.

### Viral titer measurement

Vero cells were seeded into 96-well plates. Serially diluted viruses were added to the wells. The virus titer was determined using the Reed-Muench method.^52^

### Generation of RIG-I-knockout cell lines with the CRISPR/Cas9 system

The backbone plasmids lenti-sgRNA and lenti-cas9-zeocin were obtained from GenScript Co., Ltd. (Nanjing, China). sgRNA (AATTCCCACAAGGACAAAAG) was designed and subcloned into the cas9 backbone. Lucia luciferase reporter HEK293cells were first infected with lenti-cas9-zeocin and then selected using zeocin. The stable sublines were then infected with lenti-sgRNA to specifically knock out the target genes.

### Immunofluorescence confocal

Cells grown on glass slides (Cellvis, USA) were washed with PBS and fixed with 4% paraformaldehyde (Sigma-Aldrich, P6148) in PBS for 30 min at 25 ± 2°C. They were then permeabilized with 0.2% Triton X-100 (BioFROXX, 1139ML100) in PBS for 15 min, blocked with nonfat dried milk in Tris-buffered saline (20 mM Tris-HCl, 500 mM NaCl, pH 7.4) with Tween-20 (Aladdin, T104863) (TBST) for 1 h, and incubated with primary antibodies (cGAS, 1: 1000, 79978, Cell Signaling Technology) at 4°C overnight. After washing with PBS, the cells were incubated with Alexa Fluor 488-conjugated secondary antibodies (1: 1000, Thermo Fisher Scientific, A21121) for 2 h and then stained with Hoechst 33342 (Thermo) for 5 min. Images were acquired using a Leica LSM900^®^ confocal laser-scanning microscope.

### Gene overexpression

Semiconfluent (70–80%) monolayers of stably transfected pLenti6/V5-p53 wt^62^ (Addgene #22945), R273H^62^ (Addgene #22934), KDEL-GFP^63^ (Addgene #128257), IRF3-V5^64^ (Addgene #32713), IRF3-GFP^65^ (Addgene #127663) cells were incubated in different media for 48 h.

### Cell cycle analysis

Cell cycle was conducted and analyzed according to BD BrdU Flow Kit (559619).

### Statistical analysis

Statistical analyses were performed using the GraphPad Prism 9.0 software package (GraphPad Software Inc.). Statistical analysis was conducted using a 2-tailed unpaired Student’s t-test, one-way ANOVA followed by Tukey’s multiple comparison test, or non-parametric Mann–Whitney U test. Statistical significance was set at *P* < *0.05*. Survival curves were plotted using the Kaplan–Meier method (log-rank test). Data of IHC, Western Blot and Immunofluorescence were analyzed by Image J.

### Supplementary information

Supplementary Figure 1-8, Supplementary Table 1

## Data Availability

All research data supporting the findings of this study are available upon reasonable request by readers.
