# Supplementary material for: Non-small cell lung cancers (NSCLCs) oncolysis using coxsackievirus B5 and synergistic DNA-damage response inhibitors
Source: Signal Transduct Target Ther. 2023 Sep 25;8:366. doi: 10.1038/s41392-023-01603-4 (PMC10518312; doi:10.1038/s41392-023-01603-4)
Supplement: Supplementary file 1 — Supplementary Figure 1-8, Supplementary Table 1 [file 41392_2023_1603_MOESM1_ESM.docx]

Supplementary Materials for

# NSCLC oncolysis using coxsackievirus B5 and synergistic DNA-damage response inhibitors

Bopei Cui, Lifang Song, Qian Wang, Kelei Li, Qian He, Xing Wu, Fan Gao, Mingchen Liu, Chaoqiang An, Qiushuang Gao, Chaoying Hu, Xiaotian Hao, Fangyu Dong, Jiuyue Zhou, Dong Liu, Ziyang Song, Xujia Yan, Jialu Zhang, Yu Bai, Qunying Mao, Xiaoming Yang, Zhenglun Liang

# Correspondence to:

[maoqunying@126.com](mailto:maoqunying@126.com), [yangxiaoming@sinopharm.com](mailto:yangxiaoming@sinopharm.com), [lzhenglun@126.com](mailto:lzhenglun@126.com)

This file includes:

**Supplementary** Fig. 1 to 8

**Supplementary** Table 1

**Supplementary Fig. 1**


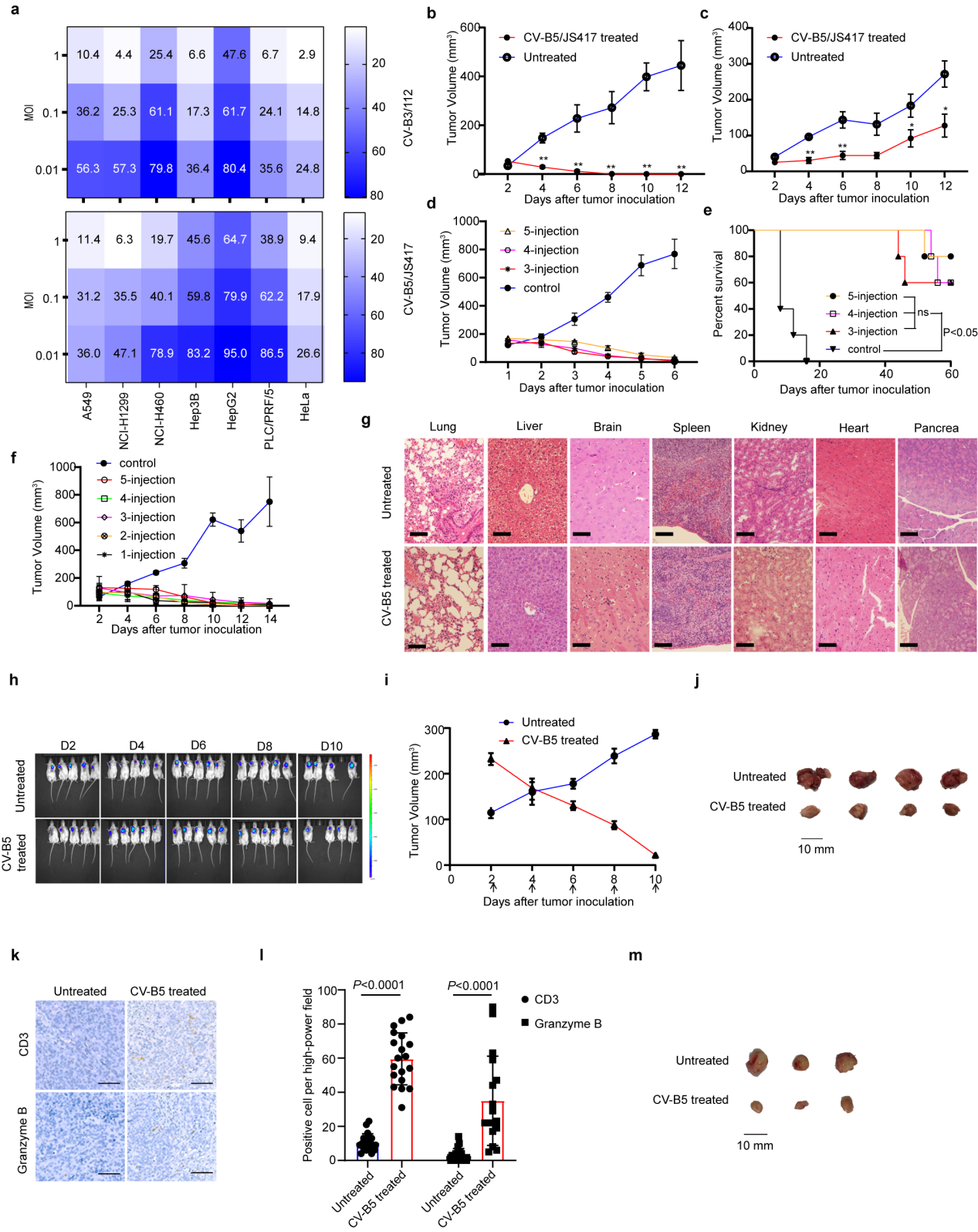


**Supplementary Fig. 1**

Oncolytic effects of CV-B3/112 and CV-B5/JS417 *in vitro* and other evidence of anti-tumor effects for CV-B5 *in vivo*. Related to Fig. 1.

(a) A549, NCI-H1299, NCI-H460, Hep3B, HepG2, PLC/PRF/5, HeLa were infected with CV-B3/112, CV-B5/JS417 at an MOI of 1, 0.1, 0.01 for 48 h. Cell viability was assessed by CCK8 assay.

(b-c) NCI-H1299 (n = 5, b) and A549 (n = 5, c) were subcutaneously injected into the right flanks of BALB/c nude mice. Each mouse received 5 doses of CV-B5/JS417 intratumorally. Tumor volumes are expressed as mean ± SEMs. *, *P* < 0.05; **,*P* < 0.01. One-way ANOVA was used to analyze data.

(d-e) NCI-H1299 (n = 5) was subcutaneously injected into the right flanks of BALB/c nude mice. Each group separately received 5/4/3 doses of CV-B5/F intratumorally when the diameter of tumor reached 7–8 mm. Tumor volumes are expressed as mean ± SEMs. *, *P* < 0.05; **, *P* < 0.01. Kaplan–Meier survival analyses were shown for CV-B5/F-treated mice.

(f) NCI-H1299 (n = 5) was subcutaneously injected into the right flanks of BALB/c nude mice. Each group separately received 5/4/3/2/1 doses of CV-B5/F intratumorally when the diameter of the tumor reached 4–5 mm. Tumor volumes are expressed as mean ± SEMs. *, *P* < 0.05; **, *P* < 0.01.

(g) Lung, liver, brain, spleen, kidney, heart, and pancreas were dissected from mice in Fig. 1 (B) for H&E staining. Scale bar, 100 μm

(h-j) Each mouse received five doses of CV-B5/F intratumorally and the volumes were recorded. The bioluminescent images of humanized mice were shown in (h); volumes were shown in (i); anatomized tumors were graphically shown in (j); IHC for CD3 and granzyme B was shown in (k) and (l). One-way ANOVA was used to analyze data (n = 20).

(m) Anatomized tumor for PDX model.


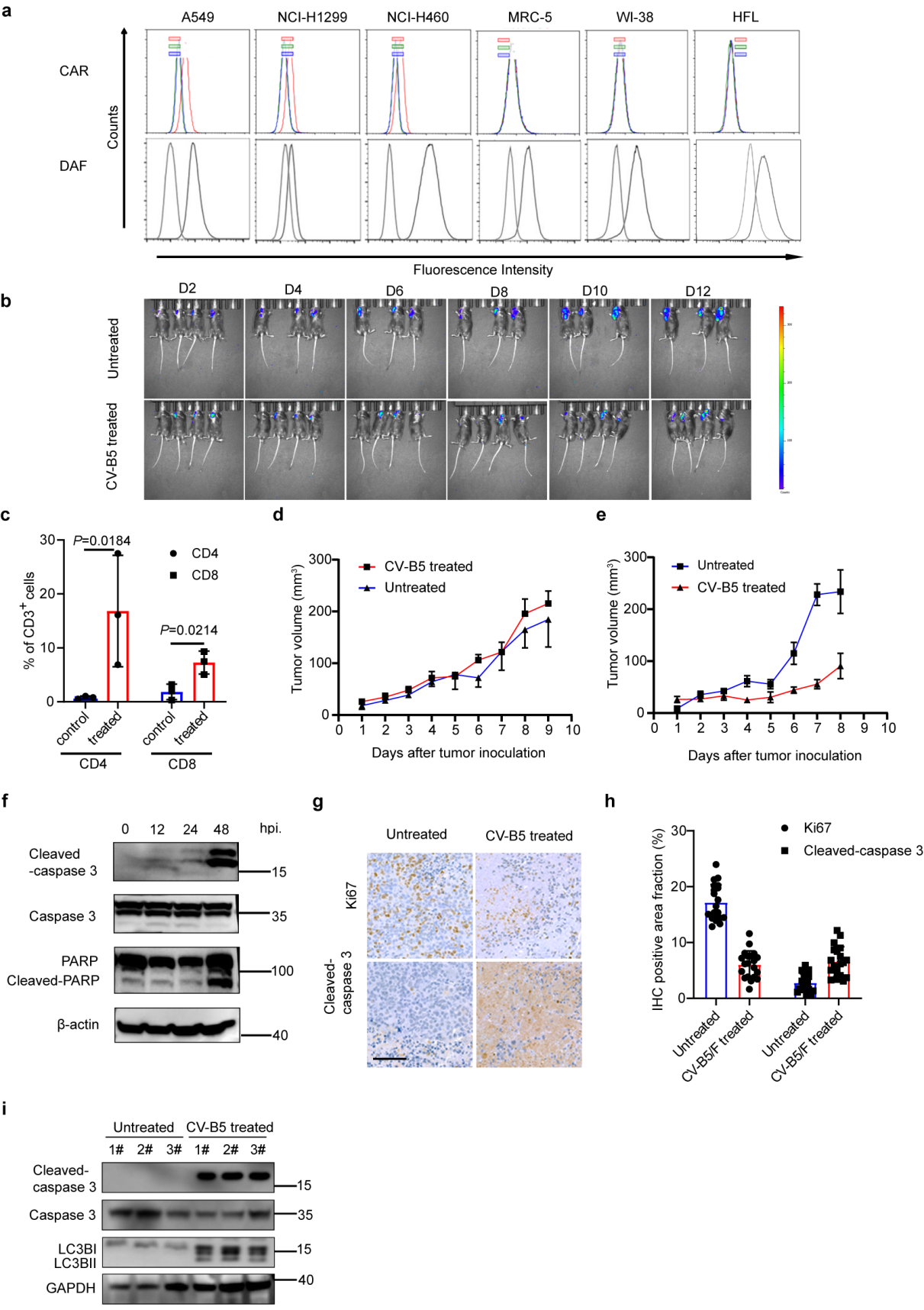


**Supplementary Fig. 2**

CV-B5/F-mediated cell death relied on the expression of a specific receptor. Related to Fig. 2.

(a) Flow cytometry analysis (FCA) of CAR and DAF on NSCLC cells and normal lung (MRC-5, WI-38, and HFL). For CAR, red peaks indicated the positive cells; green peaks indicated the negative cells; blue peaks indicated the isotype.

(b-c) The Bioluminescent images of mice in Fig. 2 (h) was shown in (b). CD4^+^ and CD8^+^ T cell in LLC-CAR xenografts were analyzed by FCA (n = 4).

(d-e) CT26.WT (d) or CT26.WT-CAR (e) were subcutaneously injected into the right flanks of BALB/c mice. Each mouse received 5 doses of CV-B5/F or with MEM intratumorally. Tumors were measured every day (n = 5).

(f) Immunoblots of cleaved-caspase 3 and cleaved-PARP after infection with CV-B5/F (MOI = 0.01) for 0, 12, 24, and 48 h in NCI-H460 cells.

(g-h) IHC of Ki67 and cleaved-caspase 3 in tumors of humanized mice in Fig. 1 (l). One-way ANOVA was used for analysis (n = 20).

(i) Immunoblots of cleaved-caspase 3 and LC3B in tumors of PDX model in Fig. 1 (n).


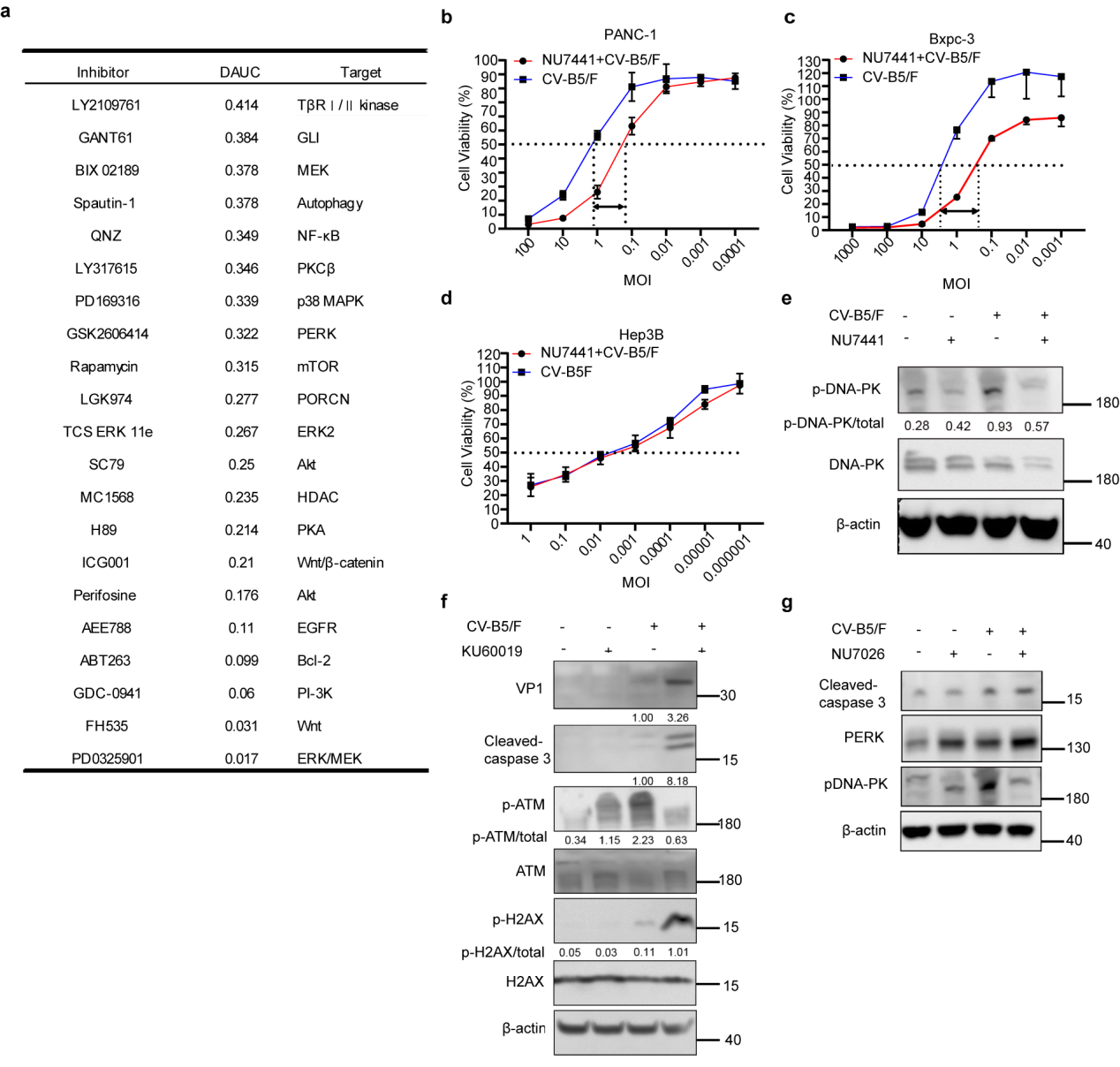


**Supplementary Fig. 3**

Synergistic effects of NU7441 and KU60019 for inducing cell death. Related to Fig. 3.

(a) DAUCs for other drugs in Fig. 3 (a).

(b-d) PANC-1 (b), Bxpc-3 (c) and Hep3B (d) were treated with escalating titers of CV-B5/F with or without 1 μM NU7441 for 48 hours. EC_50_ shifts were shown.

(e) NCI-H460 was treated with 1 μM NU7441, 0.01 MOI CV-B5/F, or a combination for 24 h. Immunoblots of p-DNA-PK were detected.

(f) NCI-H460 was treated with 1 μM KU60019, 0.01 MOI CV-B5/F, or a combination for 24 h. Immunoblots of p-ATM, p-H2AX and VP1 of CV-B5/F and cleaved-caspase 3 were detected.

(g) NCI-H460 cells were treated with 1 μM NU7026 (another inhibitor for DNA-PK), 0.01 MOI CV-B5/F, or a combination for 24 h. Immunoblots of cleaved-caspase 3, PERK and p-DNA-PK were analyzed.


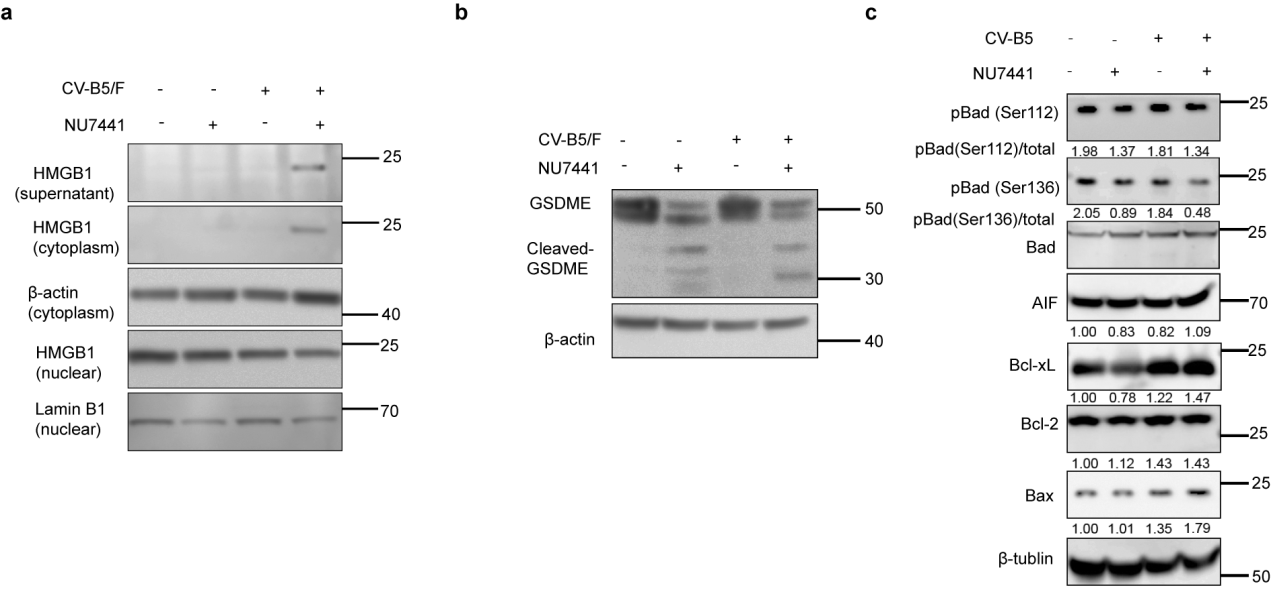


**Supplementary Fig. 4**

**Related cell death pathways.**

(a) NCI-H460 cells were treated with 1 μM NU7441, 0.01 MOI CV-B5/F, or a combination for 24 h. Immunoblots of HMGB1 in supernatant, cytoplasm, and nuclear were separately analyzed.

(b) NCI-H460 cells were treated with 1 μM NU7441, 0.01 MOI CV-B5/F, or a combination for 24 h. Immunoblot of GSDME was analyzed.

(c) NCI-H460 cells were treated with 1 μM NU7441, 0.01 MOI CV-B5/F, or a combination for 24 h. Immunoblots of a panel of pro-apoptosis and anti-apoptosis proteins were detected.


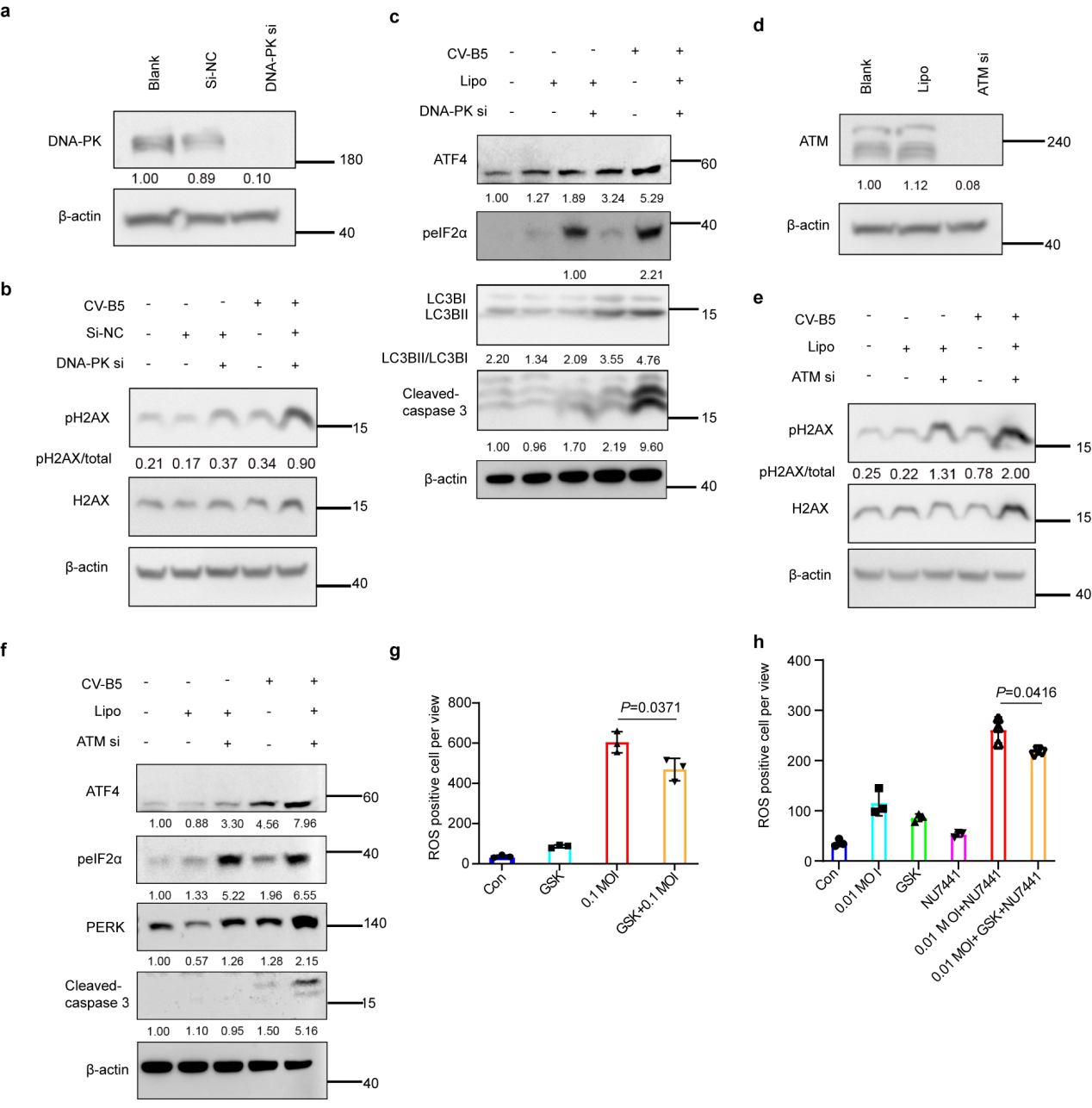


**Supplementary Fig. 5**

SiRNA targeting DNA-PK or ATM can also synergize with CV-B5/F to induce ERS-related cell death, and ROS scavengers can inhibit PERK pathway and viral replication. Related to Fig. 4.

(a-c) Knock-down DNA-PK (a) can upregulate the phosphorylation of H2AX (b) in NCI-H460 cell, and activate cell death by detecting ATF4, p-eIF2α, LC3B, and cleaved-caspase 3 by immunoblotting (c).

(d-f) Knock-down ATM (d) can upregulate the phosphorylation of H2AX (e), and activate cell death by detecting ATF4, p-eIF2α, PERK and cleaved-caspase 3 by immunoblotting (f).

(g) NCI-H460 cell was treated with 5 μM GSK2606414, 0.1 MOI CV-B5/F, or a combination for 24 h. Reactive oxygen species (ROS) of each were detected (n = 3). One-way ANOVA was used to analyze data.

(h) NCI-H460 was treated with 1 μM NU7441, 5 μM GSK2606414, 0.01 MOI CV-B5/F, or a combination of NU7441 and CV-B5/F with or without 5 μM GSK2606414. Reactive oxygen species (ROS) of each were detected (n = 3). One-way ANOVA was used to analyze data.


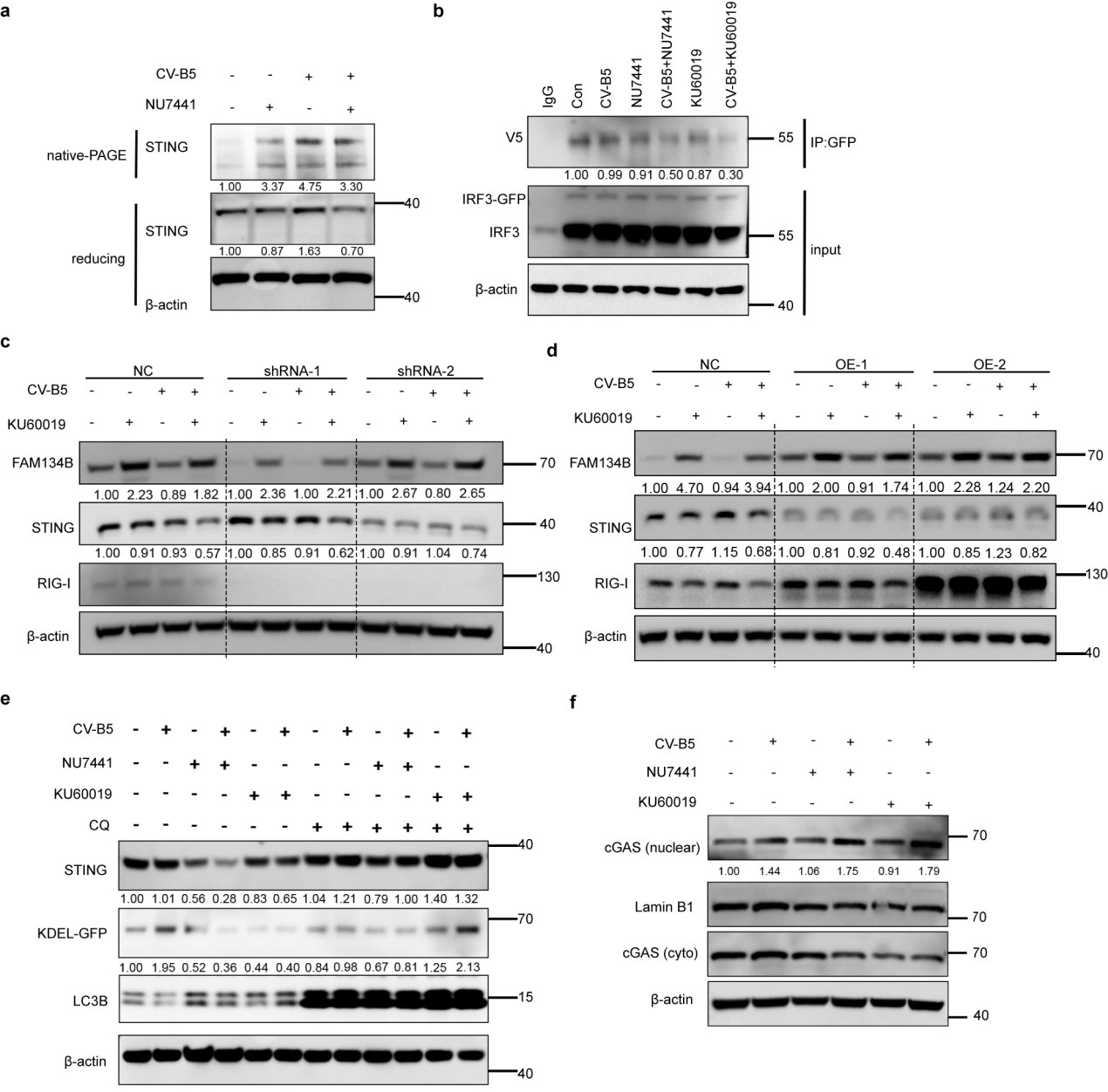


**Supplementary Fig. 6**

CV-B5/F activated cGAS-STING pathway, and the activation can be inhibited by exacerbated ERphagy induced by the combination of inhibitors and CV-B5/F. Related to Fig. 6.

(a) NCI-H460 was treated with NU7441, CV-B5/F, or a combination. STING was detected by Western blot assay under reducing and native PAGE.

(b) NCI-H460 was overexpressed with IRF3-V5 and IRF3-GFP. Dimerization of IRF3 was detected by Co-IP.

(c) NCI-H460 cells knocked down with shRNA for RIG-I were treated with 1 μM KU60019, 0.01 MOI CV-B5/F, or a combination for 24 h. RIG-I, FAM134B and STING were determined by western blot. Gray values of NC, shRNA-1 and shRNA-2 were compared separately.

(d**)** NCI-H460 cells overexpressing RIG-I were treated with 1 μM KU60019, 0.01 MOI CV-B5/F, or a combination for 24 h. RIG-I, FAM134B and STING were determined by western blot. Gray values of NC, OE-1 and OE-2 were compared separately.

(e) NCI-H460 cells overexpressing KDEL-GFP were treated with 1 μM NU7441/KU60019, 0.01 MOI CV-B5/F, or a combination with/without 100μm CQ for 24 h. STING, KDEL-GFP and LC3B were detected by western blot assay.

(f) NCI-H460 cells were treated with 1 μM NU7441/KU60019, 0.01 MOI CV-B5/F, or a combination. Immunoblots of cGAS in nuclear and cytoplasm were separately analyzed by western blot assay.


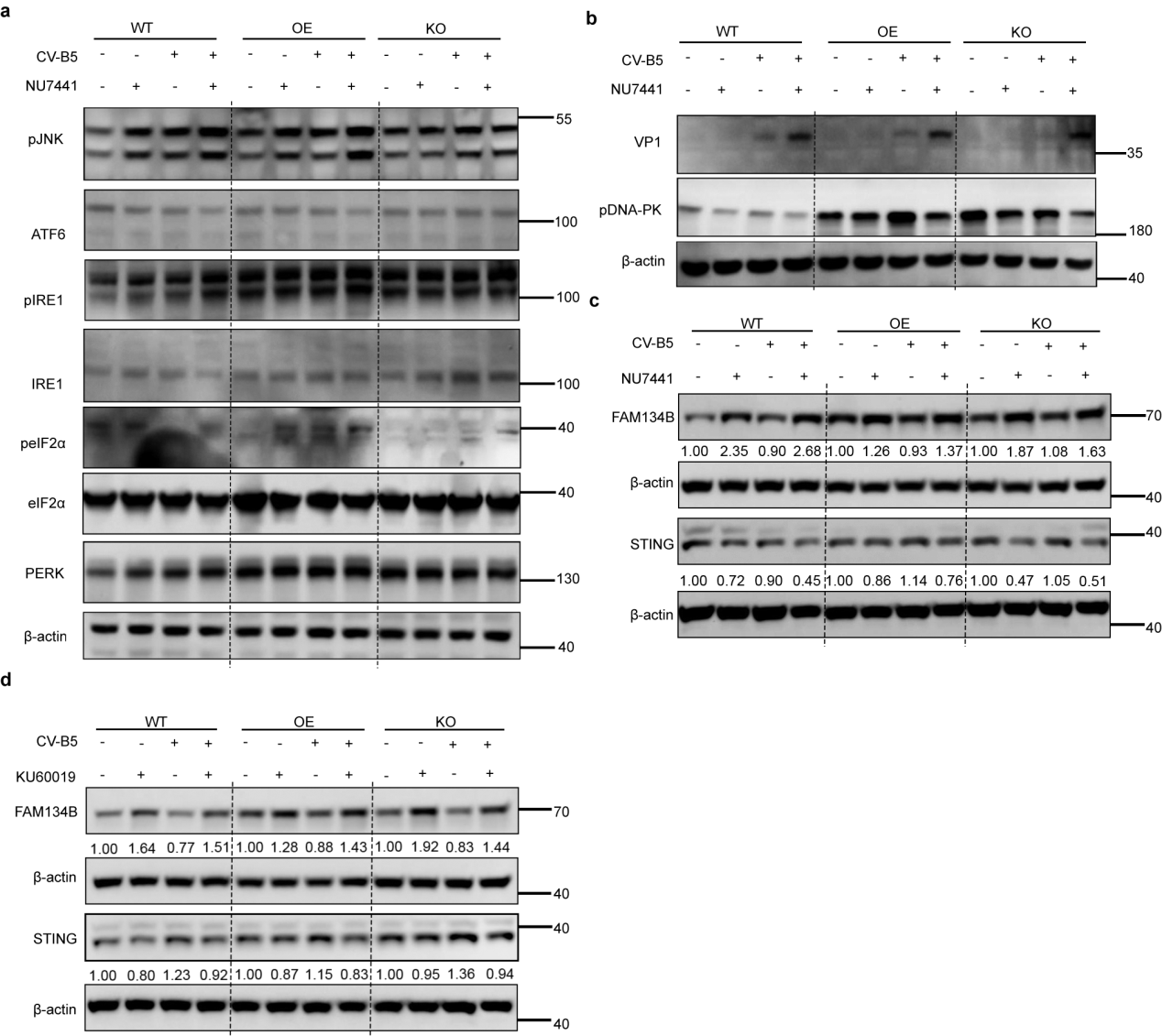


**Supplementary Fig. 7**

STING degradation and ERphagy were detected in HEK293 cell after treated with inhibitor and CV-B5/F.

(a-b) HEK 293/WT, HEK 293/RIG-I over-expression and HEK 293/RIG-I knock-out cells were treated with 1 μM NU7441, 0.01 MOI CV-B5/F, or a combination for 48 h. Immunoblots of p-JNK, ATF6, p-IRE1, p-eIF2α, PERK, VP1, and p-DNA-PK were analyzed.

(c-d) HEK 293/WT, HEK 293/RIG-I OE and HEK 293/RIG-I KO cells were treated with 1 μM NU7441 (c) or KU60019 (d), 0.01 MOI CV-B5/F, or a combination for 48 h. FAM134B and STING were determined by western blot assay. Gray values of WT, OE and KO were compared separately.


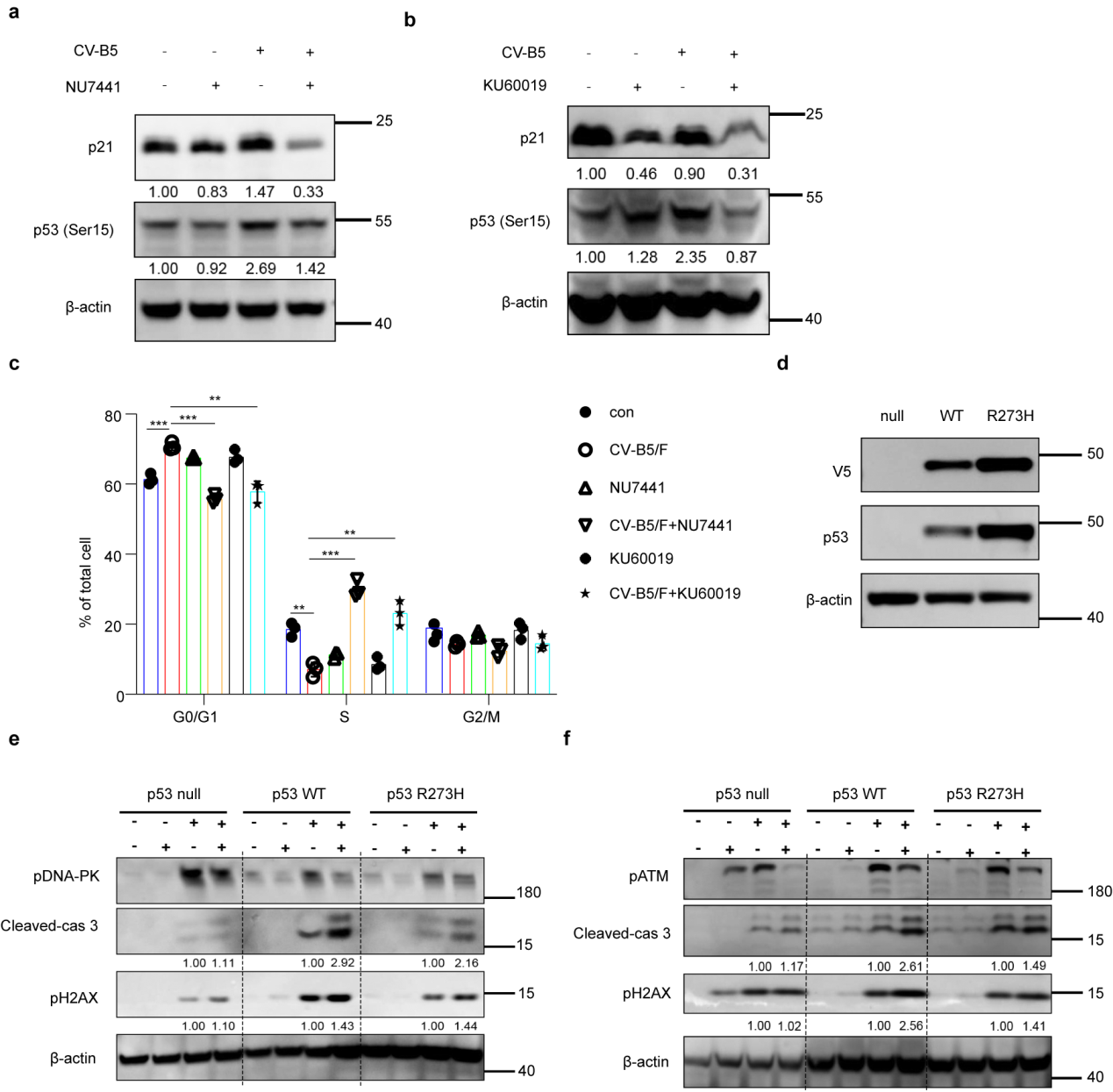


**Supplementary Fig. 8**

The synergistic effect of NU7441/KU60019 was related to p53 pathway.

(a-b) NCI-H460 cells were treated with 1 μM NU7441 (a) or 1 μM KU60019 (b), 0.01 MOI CV-B5/F, or a combination for 24 h. Immunoblots of p21 and p-p53 (Ser15) were analyzed.

(c) NCI-H460 cells were treated with 1 μM NU7441 or 1 μM KU60019, 0.01 MOI CV-B5/F, or a combination for 24 h. Cell cycle was analyzed (n = 3). One-way ANOVA was used to analyze data. **P*<0.05, ***P*<0.01, ****P*<0.001, *****P*<0.0001

(d-f) NCI-H1299 cell was overexpressed with p53 WT plasmid or p53 R273H mutant plasmid, and treated with 1 μM NU7441 (e) or 1 μM KU60019 (f), 0.01 MOI CV-B5/F, or a combination for 24 h. Immunoblots of p-DNA-PK/p-ATM, p-H2AX and cleaved caspase 3 were analyzed. Gray values of p53 null, WT and R273H were compared separately.

**Supplementary Table 1**

Basic information for viruses

| **GenBank No.** | **Name** | **Virus** | **Passage Cell** | **LgTCID_50_(/mL)** |
| --- | --- | --- | --- | --- |
| AF114383 | Faulkner | CV-B5 | LLC-MK2 | 7.5 |
| KY303900 | JS417 | CV-B5 | LLC-MK2 | 7.8 |
| M88483 | Nancy | CV-B3 | Vero | 7 |
| KP036480 | 112 | CV-B3 | Vero | 8 |
| AY421764 | Gdula | CV-A6 | RD | 7.2 |
